# Development of the W-PREV Model: Integrating HIV/STBBI Prevention and Women's Sexual and Reproductive Healthcare Using an Intersectional Women-Centered Approach

**DOI:** 10.1177/23259582261447168

**Published:** 2026-05-08

**Authors:** Kyla Gibson, Amy Ly, V Logan Kennedy, Angela Underhill, Stephanie Smith, Emily Bear, Cara Spence, Wangari Tharao, Molly Bannerman, Asli Mahdi, Ananya Inaganti, Rsha Soud, Ashley Lacombe-Duncan, Mona Loutfy

**Affiliations:** 1 Research and Innovation Institute, 7985 Women's College Hospital, Toronto, Canada; 2 College of Social and Applied Human Sciences, 3653 University of Guelph, Guelph, Canada; 3 Wellness Wheel Clinic, Regina, Canada; 4 College of Medicine, 7235 University of Saskatchewan, Saskatoon, Canada; 5 Women's Health in Women's Hands, Toronto, Canada; 6 Women and HIV/AIDS Initiative, Toronto, Canada; 7 1259 School of Social Work, University of Michigan, Ann Arbor, USA; 8 Institute of Health Policy, Management and Evaluation, Dalla Lana School of Public Health, University of Toronto, Toronto, Canada; 9 Maple Leaf Medical Clinic, Toronto, Canada

**Keywords:** Sexual and reproductive health, HIV prevention, STBBI prevention, women, implementation science, model of care, women-centred HIV care

## Abstract

**Introduction:**

Women, both cisgender and transgender, experience persistent inequities in sexual and reproductive health (SRH) due to sexism, transphobia, racism, and systemic discrimination. In Canada and globally, these intersecting inequities are exacerbated by limited, biomedical, and colonial models of care that overlook women's needs, leaving them underserved in HIV and sexually transmitted and blood-borne infection (STBBI) prevention. Our study describes the development of the Women-Centred Prevention (W-PREV) Model, designed to integrate HIV/STBBI prevention and SRH through an intersectional, women-led approach.

**Methods:**

Guided by the implementation science Knowledge-to-Action Framework, we adapted the existing Women-Centred HIV Care (WCHC) Model to address HIV/STBBI prevention and SRH needs for women. The development process included a rapid scoping review, environmental scan, and stakeholder interviews with women, clinicians, and service providers in two Canadian provinces, Ontario and Saskatchewan.

**Results:**

Findings highlighted that structural barriers such as housing insecurity, stigma, and systemic racism often overshadow women's ability to prioritize HIV/STBBI prevention and SRH. The resulting W-PREV Model addresses these realities by integrating HIV/STBBI prevention within six interrelated domains: SRH care, gender-specific care, mental health care, substance use and harm reduction, social connection and peer support, and individual capacity building. The model's trauma- and violence-aware, person-centred, and culturally responsive foundation ensures prevention is accessible, relevant, and empowering across the life course. W-PREV is distinct in its focus on HIV/STBBI prevention and early intervention, integrating SRH, peer support, and outreach within community and clinical settings.

**Conclusions:**

The W-PREV Model provides a comprehensive, women-centred framework that bridges clinical and community settings to support personalized HIV/STBBI prevention and SRH self-care. By grounding prevention in women's lived experiences and peer support, W-PREV promotes equitable, holistic, and sustainable care for women and gender diverse people in Canada.

## Plain Language Summary Title

Developing a women-centred approach to HIV/STBBI prevention and sexual and reproductive health care

**Plain Language Summary:**

**Why was this research done?**

HIV and sexually transmitted and blood-borne infections (STBBI) rates are rising in women in Ontario and Saskatchewan. Women persistently face gaps in sexual and reproductive health care and HIV/STBBI prevention due to intersecting social and structural inequities. The Women-Centred Prevention (W-PREV) Model of Care was created to better support women who are often overlooked in traditional health systems. It uses a women-centred, trauma-informed approach that integrates clinical and community services to empower women, increase HIV/STBBI prevention access, and improve health outcomes.

**How does this research contribute to the field?**

This research describes the methods used to develop an intersectional, women-centred approach to HIV and STBBI prevention. It offers a practical, evidence-based model of care that can be used in different settings to improve health equity for women. It also shows that clinical and community services need to work together to better support women who face barriers to HIV/STBBI prevention and sexual and reproductive health care.

**What are your research's implications for theory, practice, or policy?**

This work represents a step towards responding to the unmet sexual and reproductive health needs of women in Canada. The W-PREV Model is constructed so it may be tailored to diverse care contexts. It provides a practical framework that can be used by healthcare providers, outreach workers, and community organizations to improve care for women and gender diverse people. Our team is continuing this work by testing and implementing the W-PREV Model through a clinical and community-based program in Ontario and Saskatchewan.

## Introduction

Globally, women experience systemic and gender-based inequities that contribute to problematic patterns of violence and poor sexual and reproductive health (SRH).^1–3^ While Canada is committed to ending the HIV epidemic, HIV diagnoses increased nearly a quarter (24.9%) between 2021 and 2022, with the national rate jumping from 3.8 to 4.7 per 100,000 population.^4^ Cisgender (cis) women represent 25.3% of all people living with HIV in Canada, but accounted for 35.5% of all new infections in 2022.^5^ Also, for the first time, heterosexual sex became the leading route of HIV transmission in 2022.^4^

Provincially, both Ontario and Saskatchewan face distinct but pressing challenges. Women in Saskatchewan bear the brunt of new HIV infections in Canada.^4,6^ In 2019, cis women represented about half of new HIV diagnoses in Saskatchewan.^4,6^ In 2018, among reproductive-age cis women in Saskatchewan, the HIV incidence was nearly four times the overall Canadian rate, highlighting a striking difference in HIV prevention needs.^7^ Since 2020 in Ontario, cis women have also comprised a growing proportion of new HIV infections.^4^ In 2021, young cis women (age 15-19) were at higher risk of HIV acquisition than their male counterparts.^4,8^ Globally, transgender (trans) women have a significantly higher HIV prevalence than trans men (19.9% vs 2.6%), with both groups disproportionately affected compared to the general population.^9^

Moreover, rates of sexually transmitted infections (STIs) are also rising in Canada. Between 2012 and 2019, rates in Canada for chlamydia and gonorrhea increased by 22.0% and 151.0%, respectively.^10^ In 2021, cis women represented 59.0% of reported chlamydia cases and 37.0% of reported gonorrhea cases.^10^ Ontario and Saskatchewan account for a significant portion of new chlamydia and gonorrhea cases. In Ontario, from 2015 to 2022, young cis women (age 13-19) represented the majority of new chlamydia and gonorrhea cases in comparison to young cis men (74.6% vs 25.4% and 61.2% vs 38.8%, respectively).^11^ The resurgence of infectious syphilis is particularly alarming. Between 2017 and 2021, the rate of infectious syphilis increased by 729.0% in cis women and only 96% in cis men.^12^ Rising infections among reproductive-age people have also driven a 1271.0% increase in congenital syphilis cases during the same period.^12^ Saskatchewan continues to experience some of the highest rates of early congenital syphilis in the country, with 189.7 cases per 100,000 live births in 2022, compared to 32.7 cases nationally and 20.0 cases per 100,000 in Ontario.^13^

Women face heightened vulnerability to HIV and other sexually transmitted and blood-borne infections (STBBIs) due to intersecting social, structural, and biological factors.^14^ Intersectionality Theory is a framework used to understand how systems of oppression compound and interlock to perpetuate inequality, including racism, sexism, ableism, and classism.^15–17^ It further suggests that not all forms of inequality are experienced in the same way.^15,16^ The theory compels us to build holistic solutions that acknowledge how various forms of inequality exacerbate each other and are shaped by all parts of one's identity, rather than a sum of separate parts.^15^ In HIV prevention, the Intersectionality Theory examines how the intersection of (1) health conditions (eg, HIV, mental illness, substance use disorder); (2) positions and identities (eg, race, gender, sexual orientation, immigration status); and (3) behaviors (eg, substance use, sex work) shape experiences of stigma and influence HIV risk.^18^ Systemic racism, settler colonialism, and social marginalization are well-documented drivers of HIV and STBBI acquisition, contributing to disproportionate rates among racialized, Indigenous, and newcomer women in Canada.^19,20^ These inequities are even more pronounced for trans and gender diverse women.^21^ Many women navigate complex power dynamics, such as transactional or survival sex, that may limit their ability to negotiate safer sexual practices, thereby increasing their HIV/STBBI exposure risk.^14^ Lacombe-Duncan and Olawale found that high rates of violence (eg, bullying, familial rejection) in childhood and early adulthood experienced by trans women increased HIV vulnerability.^22^ Women who use drugs are at further risk of acquiring HIV and blood-borne infections through intravenous drug use.^23^ Biological factors also contribute to an increased risk for HIV/STBBI acquisition for women in comparison to men when engaging in penetrative vaginal intercourse.^24–26^ The risk of gonorrhea and HIV transmission from males to females during vaginal intercourse is nearly 3-fold and 2-fold greater than females to males, respectively.^24–26^

Despite the availability of effective HIV prevention tools, uptake remains disproportionately higher among gay, bisexual, and other men who have sex with men (gbMSM) compared to women in Canada.^27^ This imbalance has contributed to declining HIV incidence among men, while rates among women have remained stagnant or increased.^20,28^ Current prevention strategies continue to fall short for women because they inadequately address the root causes of HIV and STBBI acquisition. For instance, testing can only interrupt transmission if treatment is accessed and adhered to – both of which depend on factors such as drug coverage, socioeconomic stability, and continuity of care.^29,30^ Similarly, condom use, often framed as a gender-neutral prevention method, is constrained by social and gendered power imbalances. Condoms remain largely male-controlled, and expectations that women should purchase, carry, and insist on their use conflict with societal norms that construct women as passive participants in sexual relationships.^31,32^

Pre-exposure prophylaxis (PrEP), one of the few self-controlled prevention tools, has not been widely promoted or researched for women in Canada.^33–36^ Popovic et al reported that almost all (98.0%) of PrEP users in nine Canadian provinces between 2018 and 2021 were male.^27^ Additionally, PrEP requires a woman and/or her care provider to be aware of PrEP, have access to privacy for acquiring a prescription, storing and taking medication, and believe she is at risk of HIV.^37^ These barriers are all amplified by HIV-related stigma.^37^ Gaps in knowledge dissemination also cause many women to be unaware of alternatives to PrEP, like HIV post-exposure prophylaxis (PEP) and PEP-in-pocket (PIP).^38,39^ Similarly, options like DoxyPEP for bacterial STI prevention are overlooked for women, as these medications also predominantly focus on gbMSM.^40^ These tools represent a universal approach to HIV/STBBI prevention**.** However, as reflected in the disappointing PrEP uptake in women, our system fails to acknowledge that women experience HIV/STBBI risk differently than men, due to syndemic health and structural vulnerabilities.^14,27^

This lack of progress is further exacerbated by the minimal integration of care addressing the root causes of HIV/STBBI acquisition into HIV/STBBI prevention. For example, Salway *et al* found that 39% of clients at a sexual health clinic in a large Canadian urban center reported a need for mental health or substance-related care, but siloed services created gaps in accessing this care along with HIV/STBBI services.^41^ Further, a study in a major Canadian city highlighted that young female sex workers felt that sexual healthcare providers would be uninformed about their social experiences and had concerns about confidentiality and being judged.^42^ Other gaps include a lack of comprehensive care for conception and contraception needs.^42,43^ Studies suggest that women living with HIV are less likely to receive pre-conception counseling and associated costs, clinic and procedure disintegration, and intersecting forms of stigma create barriers to accessing desired contraception methods.^42,43^

Therefore, integrated, women-centred prevention approaches are urgently needed. Building on findings from the Canadian HIV Women's Sexual and Reproductive Health Cohort Study (CHIWOS) and a women-centred care model used in Vancouver, the Women-Centred HIV Care (WCHC) Model was developed as an evidence-based, holistic framework addressing the needs of women living with HIV.^36,44–46^ The WCHC Model was found to be acceptable among cis and trans women and individuals with transfeminine experience.^45,47^ Depicted as a house, the model places trauma- and violence-aware care as its foundation, person-centred and social determinant–focused care on the first floor, and integrated HIV, women's and mental health on the second, and finally peer support and leadership as the roof.^44^

In this article, we describe the adaptation of the WCHC Model into a prevention-focused framework using an implementation science approach guided by the Knowledge-to-Action Framework.^48^ Through a rapid scoping review, environmental scan, and qualitative stakeholder interviews, we developed the Women-Centred Prevention (W-PREV) Model—a holistic, intersectional model of HIV/STBBI prevention and SRH care. The W-PREV Model delivers trauma- and violence-aware, person-centred, and culturally sensitive care that integrates SRH care and services, mental health and substance use care, and peer and individual support to address women's unique needs. Grounded in empirical evidence and lived experience, the W-PREV Model aims to enhance HIV/STBBI prevention and promote SRH and mental well-being among women and gender diverse people in Canada.

## Methods

### Framework

**Figure 1.**
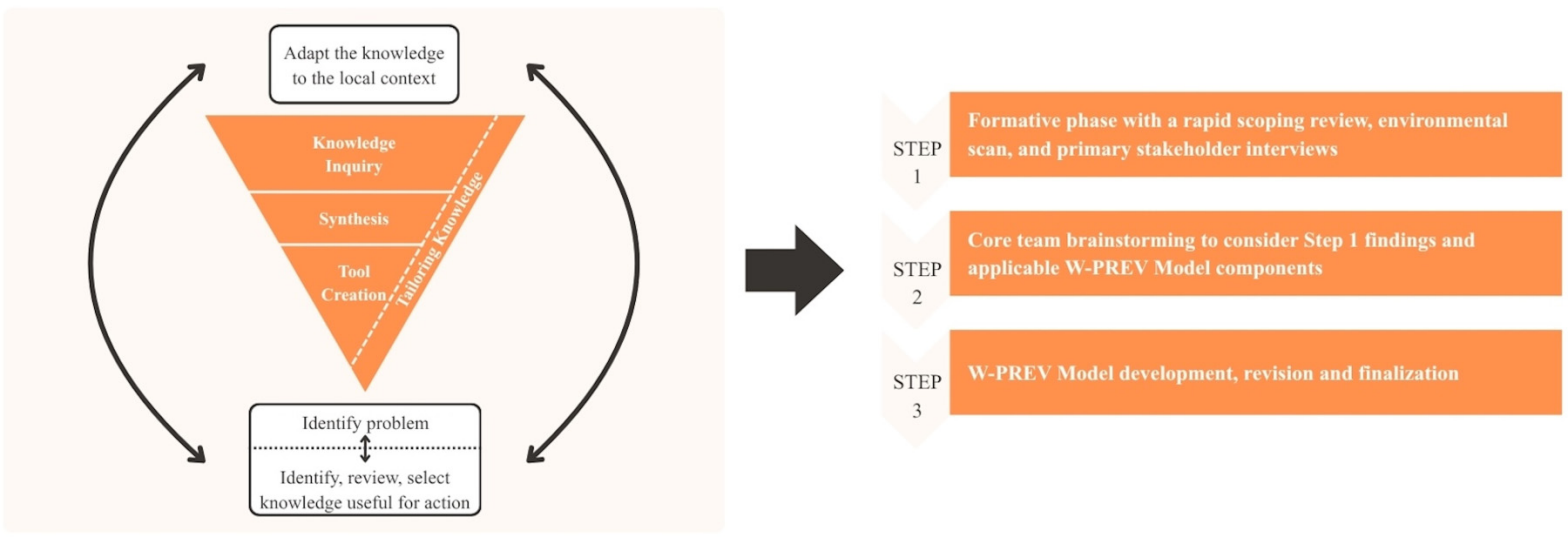
Packaging of five phases of the knowledge-to-action framework into three care model development steps.

We drew on Graham *et al*'s Knowledge-to-Action Framework to guide the translation of knowledge into actionable tools to influence health outcomes.^48^ This framework contains three knowledge creation phases and seven action cycle phases.^48^ Consistent with the approach used to develop the WCHC Model, we applied the three knowledge creation phases: (1) knowledge inquiry, (2) knowledge synthesis, and (3) product development; and two of the seven action cycle phases: (1) identifying the problem and reviewing and selecting knowledge and (2) adapting knowledge to the local context.^44,48^ These five phases were then packaged into three care model development steps^44^: (1) a formative phase with a rapid scoping review, environmental scan, and primary stakeholder interviews (knowledge inquiry and knowledge synthesis), (2) core team brainstorming to consider Step 1 findings and applicable W-PREV Model components (review and select knowledge), and (3) W-PREV Model development, revision and finalization (adapt knowledge to local context, product development) (Figure 1). These steps were used to collect empirical evidence and lived knowledge, providing a robust knowledge base for the adaptation of the WCHC Model to the W-PREV Model. The study was conducted from June 1st, 2024, to June 1st, 2025.

## Step 1: Formative Phase with a Rapid Scoping Review, Environmental Scan, and Primary Stakeholder Interviews

### Rapid Scoping Review

Following the five-stage framework developed by Arskey and O’Malley, we conducted a rapid scoping review to identify effective and integrated HIV/STBBI prevention and SRH promotion programs for women.^49^ The review generated actionable evidence to inform the development of the W-PREV Model.^50^ The process involved defining the research question, identifying and selecting relevant studies, charting the data, and summarizing the findings. Our searches aimed to answer the question “What literature exists on HIV/STBBI prevention and SRH promotion programs and strategies for women globally?” Three members of the research team conducted the review. Screening and data extraction followed the PRISMA Extension for Scoping Reviews (PRISMA-ScR) guidelines (Appendix 1).^51^ Eligibility criteria were developed using the Population, Concept, and Context (PCC) framework.^52^

**Figure 2.**
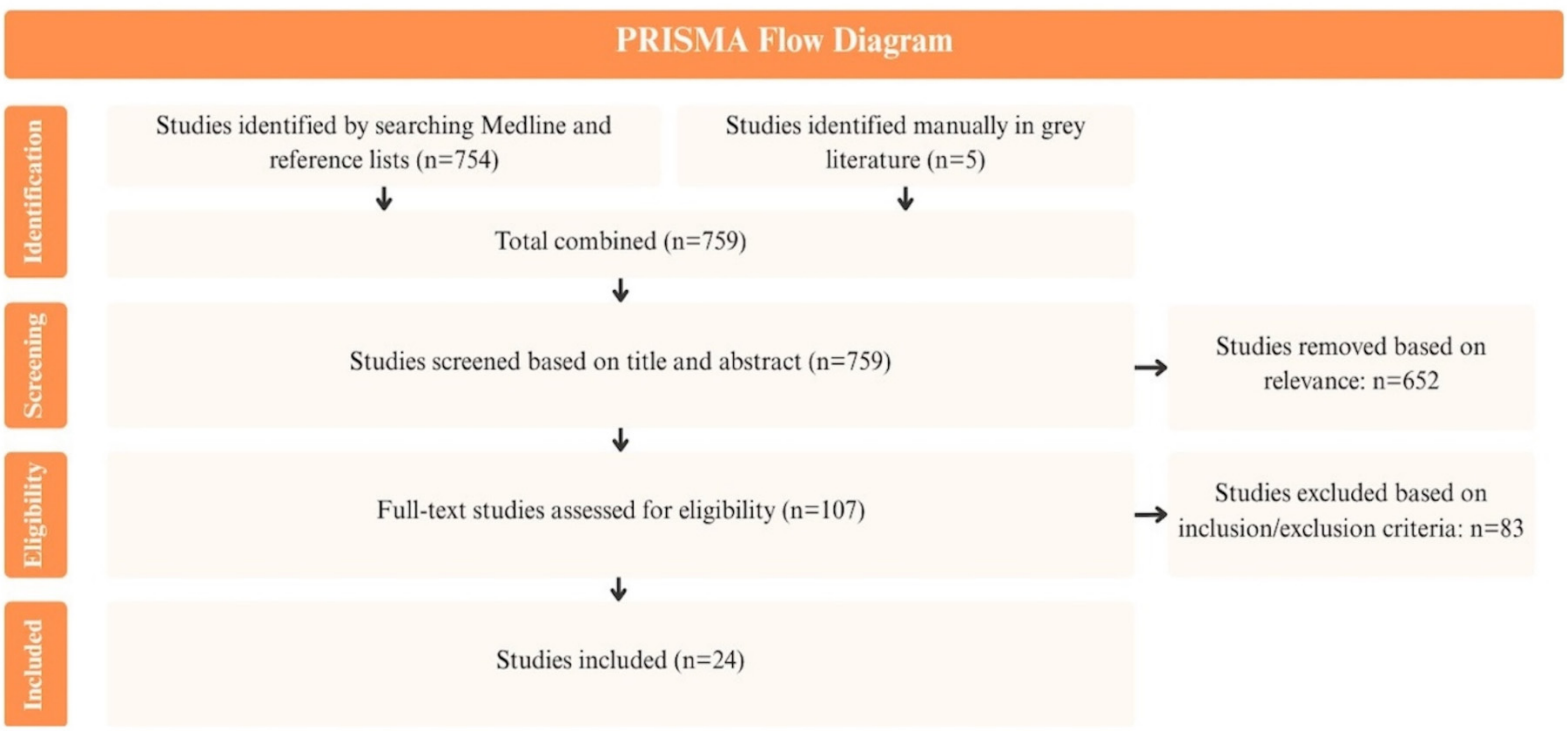
PRISMA flow diagram for rapid scoping review.

A systematic search of MEDLINE (2009–2024) was conducted with support from a health sciences librarian knowledgeable in scoping reviews.^53^ Keyword searching was conducted to ensure extensive coverage of the research topic. Search terms included “HIV,” “sexually transmitted and blood-borne infections,” “prevention,” and “women's health”.^54^ To broaden coverage, reference lists of included studies were screened using snowball sampling, and key journals were hand-searched to identify relevant grey literature. Inclusion criteria were refined iteratively as familiarity with the literature increased and focused on studies describing or evaluating HIV/STBBI prevention and SRH promotion interventions for women. Articles were required to be published in English between 2009 and 2024. Our searches identified 754 studies, and the grey literature produced an additional 5 studies. One member of the research team screened study titles and abstracts for relevance to the research question, retaining 107 studies for full-text review. Three members of the research team then independently applied the inclusion criteria to the 107 remaining studies, and 24 were included (Figure 2). Data were charted in Excel and captured the study design, population, setting, and outcomes related to HIV/STBBI prevention and SRH integration (Supplemental Table S1).

### Web-Based Environmental Scan

To assess the clinical contexts of HIV/STBBI prevention services in Ontario and Saskatchewan, we followed Inaganti *et al's* environmental scan protocol to systematically identify clinics providing these services from websites.^55^ This protocol follows Shahid and Turin's five-step framework for environment scans: (1) defining the purpose and objectives; (2) engaging key stakeholders; (3) refining the focus based on feedback; (4) gathering data comprehensively; and (5) sharing the findings.^56^ This environmental scan protocol addressed the question “what HIV/STBBI prevention options are currently available in clinical settings in Ontario and Saskatchewan, and to what extent are these options integrated with services specific to women's needs?” Detailed methods are described elsewhere.^55^

Clinics publicly listing HIV/STBBI services on their websites were identified in Ontario (*n* = 197) and Saskatchewan (*n* = 16). We then selected those explicitly offering HIV/STBBI prevention (eg, HIV self-testing, in-house testing, PrEP/PEP prescribing), resulting in 142 clinics offering HIV prevention and 166 offering STBBI prevention in Ontario and 8 clinics offering HIV prevention and 15 offering STBBI prevention in Saskatchewan. Data were extracted into a REDCap database, capturing clinic location, scope of care, and services addressing women's needs. Data were analyzed in Excel to examine the distribution, availability, and accessibility of HIV/STBBI prevention services. To contextualize clinic availability relative to regional population size, population data were obtained from publicly available provincial sources.^57,58^

### Primary Stakeholder Interviews

We conducted qualitative interviews in Ontario and Saskatchewan to identify prevention needs, service gaps, and implementation considerations and to co-develop care model components responsive to community priorities. Recruitment occurred via an approved email to community-based organizations that serve women and gender diverse people with lived/living experience. These organizations then recruited participants for the primary stakeholder interviews. A separate approved email was sent to physicians/registered nurses and service providers. Eligibility was screened prior to participation. Interviews were completed (individual and group; in person or via Zoom, per participant preference) by two members of the research team. Demographic surveys were administered before the interview and were captured in REDCap. Guided by the Consolidated Framework for Implementation Research (CFIR) and the rapid qualitative analysis approach described by Nevedal *et al*, we used semi-structured guides tailored to either (1) women with lived/living experience or (2) clinicians/service providers (Appendix 2).^59^ Each guide mapped questions to relevant CFIR domains and constructs while allowing conversational flexibility. Interviews with women with lived/living experience were done separately from interviews with clinicians/service providers. All interviews were audio recorded but not transcribed.^59^ Two members of the research team took detailed notes during interviews, which were used to complete a structured summary template aligned with relevant CFIR domains and constructs. They then cross-reviewed one another's summaries and audio recordings to ensure consistency. Weekly meetings were held to compare interpretations, refine templates, and discuss emerging themes. Completed summaries were compiled into a comparative Excel matrix, enabling rapid synthesis of key barriers, facilitators, and implementation considerations that informed the W-PREV Model.

## Step 2: Core Team Brainstorming to Integrate Step 1 Findings and Applicable W-PREV Model Components

Following the completion of Step 1 activities, findings from the rapid scoping review, environmental scan, and primary stakeholder interviews were presented to the core research team during a virtual meeting. An in-depth discussion followed to propose the initial components and structure of the W-PREV Model. Components of the model were based on the elements of the WCHC Model and key messages identified in the rapid scoping review and stakeholder interviews, and quantitative evidence from the environmental scan. Through iterative discussion, the team examined how evidence and lived experience could be translated into actionable, context-specific components of the W-PREV Model.

## Step 3: W-PREV Model Development, Revision and Finalization

The first draft of the W-PREV Model was presented to external experts during a virtual meeting in early 2025 and circulated for detailed feedback. External reviewers comprised an interdisciplinary, anti-oppressive, and community-based panel to ensure that the model reflected the diverse HIV/STBBI prevention and SRH needs of women in Ontario and Saskatchewan. The model was subsequently shared at a national scientific meeting, allowing further feedback from researchers, clinicians, and community members. Suggestions from both stages of feedback were integrated into a refined version of the W-PREV Model—a trauma-informed, women-centred model of care designed to address gaps in HIV/STBBI prevention and SRH promotion for women.

### Ethical Approval and Informed Consent

The study was approved by the Women's College Hospital's Ethics Assessment Process for Quality Improvement Projects (APQIP) (APQIP #: 2024-0040-P). All participants provided written informed consent prior to enrollment in the study.

## Results

### Rapid Scoping Review

Twenty-four articles met the inclusion criteria (Supplemental Table S1). The majority of primary studies were conducted in the United States (*n* = 5), South Africa (*n* = 5), Canada (*n* = 2), and Australia (*n* = 2). Most interventions were delivered in community-based or clinical settings (eg, primary care centers, community clinics, and hospitals). Interventions were primarily targeting culturally or ethnically specific populations, including African, Caribbean, and Black, Indigenous, and South Asian women, and sexual minority groups such as lesbian, bisexual, and queer women. Participants were predominantly adolescent girls and young women, though several studies also included women across broader age ranges and populations at increased risk for HIV/STBBIs, including sex workers, women who use substances, and refugee populations. Of the included studies, 13 were quantitative, comprising 5 pre–post quasi-experimental studies, one cross-sectional study, 1 cohort study, and 6 randomized controlled trials. Four were literature reviews, 4 used mixed-methods designs, and 3 were qualitative studies, comprising 1 descriptive, 1 secondary data analysis, and 1 grounded in community-based participatory research.

**Figure 3.**
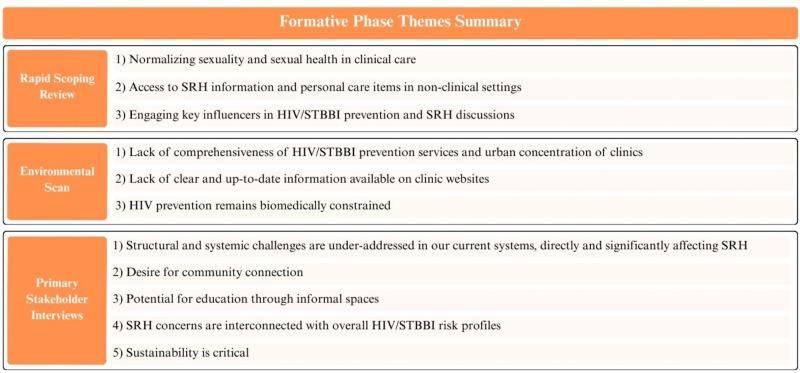
Summary of themes from rapid scoping review, environmental scan, and primary stakeholder interviews. SRH (sexual and reproductive health); STBBI (sexually transmitted and blood-borne infections).

Our analysis of the included studies revealed three overarching themes (Figure 3). First, normalizing sexuality and sexual health in clinical care has been linked with decreased fear in women in raising sexual health topics with providers.^60–62^ Integrating non-blaming and non-judgmental “safety tips” and resources within regular appointments supports the destigmatization and standardization of HIV/STBBI discussions within care, dismantles power dynamics, eases fear of disapproval, and facilitates positive sexual health practices.^60,62^ Second, access to HIV/STBBI prevention and SRH information and personal care items in non-clinical settings (eg, in community-based organizations and programs for women and girls) offers innovative opportunities to link key populations to resources and engage novel stakeholders in sexual health promotion.^63,64^ Community-based spaces have also been seen to foster trust and safety, facilitating empowerment-focused prevention.^65–67^ This approach has led to increased awareness and uptake of public health measures, like HIV self-testing.^68,69^ Third, engaging key influencers (eg, community leaders and care providers) in HIV/STBBI prevention and SRH discussions and knowledge sharing can disrupt community-level stigma to create sustained changes.^70,71^ Building social and cultural acceptance can reduce stigma and facilitate positive health behaviors.^72^ These themes indicate the need for a multi-level and integrated clinical and social approach to HIV/STBBI prevention for women.

### Web-Based Environmental Scan

The environmental scan assessed the availability and scope of HIV/STBBI prevention services in Ontario and Saskatchewan, revealing substantial variation and systemic gaps (Figure 3). The analysis showed limited comprehensiveness among clinics offering HIV/STBBI services in both provinces. While some clinics provided HIV/STBBI care, such as antiretroviral therapy, many did not offer prevention services. In Ontario, fewer than three-quarters (*n* = 142, 72.1%) of identified HIV/STBBI service clinics offered HIV prevention, compared to only half in Saskatchewan (*n* = 8, 50.0%). A higher proportion of clinics provided STBBI prevention services (*n* = 166, 84.3% in Ontario and *n* = 15, 93.8% in Saskatchewan). Laboratory-based HIV testing was the most common form of prevention offered in both provinces (Ontario: *n* = 65, 45.8%; Saskatchewan: *n* = 5, 62.5%) (Tables 1a and 1b).

**Table 1a.** Number of Clinics Offering Each HIV Prevention Service in Ontario.

Prevention Type	Toronto	Ottawa	Northern	Eastern	Central East	Central West	South West	Total Ontario
	*n* (%)	*n* (%)	*n* (%)	*n* (%)	*n* (%)	*n* (%)	*n* (%)	*n* (%)
PrEP	12 (26.1)	2 (16.7)	3 (18.8)	2 (40.0)	8 (32.0)	5 (16.7)	3 (37.5)	35 (24.6)
PEP	6 (13.0)	3 (25.0)	0 (0.0)	0 (0.0)	0 (0.0)	0 (0.0)	1 (12.5)	10 (7.0)
HIV testing (in lab)	19 (41.3)	2 (16.7)	15 (93.8)	3 (60.0)	6 (24.0)	14 (46.7)	6 (75.0)	65 (45.8)
HIV self-testing kits	10 (21.7)	1 (8.3)	3 (18.8)	4 (80.0)	3 (12.0)	3 (10.0)	1 (12.5)	25 (17.6)
HIV POC rapid testing	11 (23.9)	6 (50.0)	6 (37.5)	0 (0.0)	7 (28.0)	11 (36.7)	2 (25.0)	43 (30.3)
HIV anonymous testing	5 (10.9)	6 (50.0)	3 (18.8)	0 (0.0)	13 (52.0)	11 (36.7)	4 (50.0)	42 (29.6)
TasP	18 (39.1)	3 (25.0)	4 (25.0)	1 (20.0)	5 (20.0)	1 (3.3)	2 (25.0)	34 (23.9)
HIV prevention counseling	18 (39.1)	3 (25.0)	4 (25.0)	1 (20.0)	4 (16.0)	0 (0.0)	3 (37.5)	33 (23.2)
HIV education and outreach	14 (30.4)	0 (0.0)	6 (37.5)	1 (20.0)	1 (4.0)	1 (3.3)	4 (50.0)	27 (19.0)

Abbreviations: PrEP, pre-exposure prophylaxis; PEP, post-exposure prophylaxis; POC, point-of-care; TasP, treatment as prevention.

**Table 1b.** Number of Clinics Offering Each HIV Prevention Service in Saskatchewan.

Prevention Type	Far North	North Central West	North Central East	Saskatoon	South West	South East	Regina	Total Saskatchewan
	*n* (%)	*n* (%)	*n* (%)	*n* (%)	*n* (%)	*n* (%)	*n* (%)	*n* (%)
PrEP	0 (0.0)	0 (0.0)	0 (0.0)	2 (50.0)	0 (0.0)	0 (0.0)	0 (0.0)	2 (25.5)
PEP	0 (0.0)	0 (0.0)	0 (0.0)	0 (0.0)	0 (0.0)	0 (0.0)	0 (0.0)	0 (0.0)
HIV testing (in lab)	0 (0.0)	1 (50.0)	0 (0.0)	2 (50.0)	0 (0.0)	0 (0.0)	2 (100.0)	5 (62.5)
HIV self-testing kits	0 (0.0)	0 (0.0)	0 (0.0)	1 (25.0)	0 (0.0)	0 (0.0)	1 (50.0)	2 (25.5)
HIV POC rapid testing	0 (0.0)	0 (0.0)	0 (0.0)	2 (50.0)	0 (0.0)	0 (0.0)	0 (0.0)	2 (25.5)
HIV anonymous testing	0 (0.0)	0 (0.0)	0 (0.0)	1 (25.0)	0 (0.0)	0 (0.0)	1 (50.0)	2 (25.5)
TasP	0 (0.0)	0 (0.0)	0 (0.0)	1 (25.0)	0 (0.0)	0 (0.0)	1 (50.0)	2 (25.5)
HIV prevention counseling	0 (0.0)	0 (0.0)	0 (0.0)	0 (0.0)	0 (0.0)	0 (0.0)	0 (0.0)	0 (0.0)
HIV education and outreach	0 (0.0)	0 (0.0)	0 (0.0)	1 (25.0)	0 (0.0)	0 (0.0)	1 (50.0)	2 (25.5)

Abbreviations: PrEP, pre-exposure prophylaxis; PEP, post-exposure prophylaxis; POC, point-of-care; TasP, treatment as prevention.

Among clinics providing HIV prevention, just over half in Ontario (*n* = 87, 61.3%) and three-quarters in Saskatchewan (*n* = 6, 75.0%) offered women-specific services, such as contraception and abortion care, trauma and violence support, and peer-led or empowerment programs (Tables 2a and 2b).

**Table 2a.** Other Services at Clinics Offering HIV Prevention Services in Ontario.

Service Type	Toronto	Ottawa	Northern	Eastern	Central East	Central West	South West	Total Ontario
	*n* (%)	*n* (%)	*n* (%)	*n* (%)	*n* (%)	*n* (%)	*n* (%)	*n* (%)
Women's services	25 (54.3)	6 (50.0)	9 (56.3)	3 (60.0)	17 (68.0)	23 (76.7)	4 (50.0)	87 (61.3)
Services addressing SDH	27 (58.7)	6 (50.0)	8 (50.0)	3 (60.0)	5 (20.0)	9 (30.0)	4 (50.0)	62 (43.7)
Services for sex workers	7 (15.2)	2 (16.7)	1 (6.3)	0 (0.0)	0 (0.0)	7 (23.3)	0 (0.0)	17 (12.0)

Abbreviation: SDH, social determinants of health.

**Table 2b.** Other Services at Clinics Offering HIV Prevention Services in Saskatchewan.

Service Type	Far North	North Central West	North Central East	Saskatoon	South West	South East	Regina	Total Saskatchewan
	*n* (%)	*n* (%)	*n* (%)	*n* (%)	*n* (%)	*n* (%)	*n* (%)	*n* (%)
Women's services	0 (0.0)	1 (50.0)	0 (0.0)	3 (75.0)	0 (0.0)	0 (0.0)	2 (100.0)	6 (75.0)
Services addressing SDH	0 (0.0)	1 (50.0)	0 (0.0)	3 (75.0)	0 (0.0)	0 (0.0)	1 (50.0)	5 (62.5)

Abbreviation: SDH, social determinants of health.

Furthermore, HIV prevention services were heavily concentrated in urban centres. For instance, Toronto had 1.52 clinics per 100,000 residents offering HIV prevention, while the South West health region had only 0.45 (Table 3a).^57^ In Saskatchewan, regional disparities were even greater. The North Central West health region had 2.00 clinics per 100,000, while several northern and southern regions of the province had none (Table 3b).^58^

**Table 3a.** Clinics Offering HIV Prevention Services per 100,000 People in Ontario by Health Region.

Data Type	Toronto	Ottawa	Northern	Eastern	Central East	Central West	South West
Population^a^	3025647	1071868	819744	907777	4550794	2960636	1772950
Number of clinics	46	12	16	5	25	30	8
Per capita	0.00001520	0.00001120	0.00001952	0.00000551	0.00000549	0.00001013	0.00000451
Per 100,000	1.52	1.12	1.95	0.55	0.55	1.01	0.45

a Data adapted from a publicly available data source.^57^

**Table 3b.** Clinics Offering HIV Prevention Services per 100,000 People in Saskatchewan by Health Region.

Data Type	Far North	North Central West	North Central East	Saskatoon	South West	South East	Regina
Population^a^	38959	100138	130551	338106	136091	187662	273351
Number of clinics	0	2	0	4	0	0	2
Per capita	0.00000000	0.00001997	0.00000000	0.00001183	0.00000000	0.00000000	0.00000732
Per 100,000	0.00	2.00	0.00	1.18	0.00	0.00	0.73

a Data adapted from a publicly available data source.^58^

In addition, there was a lack of clear, up-to-date information available on clinic websites. Details regarding the range of services, eligibility criteria, and access procedures were often incomplete or inconsistent, creating possible confusion for women seeking care. Many clinic websites contained broken links or were permanently closed, especially in rural and northern regions of the provinces.

Moreover, HIV prevention remains biomedically constrained, neglecting women's diverse experiences shaped by social determinants of health (SDH). Indeed, sociocultural contexts and perception of risk influence women's uptake and adherence to biomedically-framed HIV prevention methods (eg, PrEP, PEP, testing).^14^ Among clinics offering HIV prevention, services that better acknowledge the structural and SDH, like HIV prevention counseling, education, and outreach, represented about one-fifth (*n* = 60, 19.1%) of services available in Ontario and one-tenth (*n* = 2, 11.8%) of services available in Saskatchewan. This indicates a lack of prevention efforts that can be tailored to women's needs.^73^ However, clinics in Ontario (*n* = 62, 43.7%) and Saskatchewan (*n* = 5, 62.5%) also offered programs to reduce SDH-related barriers such as mental health counseling, housing and legal support, food and income assistance, newcomer and refugee programs, employment and education services (Tables 2a and 2b).

Similar findings were observed among clinics providing STBBI prevention. Testing was the most common service (Ontario: *n* = 133, 80.1%; Saskatchewan: n = 12, 80.0%) (Supplemental Tables S2 and S3). Women's services were available in a significant proportion of these clinics (Ontario: *n* = 120, 72.3%; Saskatchewan: *n* = 12, 80.0%) (Supplemental Tables S4 and S5). Services that aim to reduced SDH-related barriers were available at less than half of clinics (Ontario: *n* = 66, 39.8%; Saskatchewan: *n* = 6, 40%). Clinic distribution was again clustered in urban and northern regions—particularly in the Northern health region (1.95 per 100,000) and Toronto (1.32 per 100,000) in Ontario, and in the North Central West health region (2.00 per 100,000) and Saskatoon (1.48 per 100,000) in Saskatchewan (Supplemental Tables S6 and S7).^57,58^

### Primary Stakeholder Interviews

Interviews were conducted with a total of 42 participants, including 24 women with lived or living experience and 18 clinicians or service providers. Most women with lived/living experience were between 40 and 50 years of age (*n* = 9, 38.0%) and also identified as people who use drugs (*n* = 16, 27.0%), sex workers or clients of sex workers (*n* = 13, 22.0%), and primarily resided in Toronto (*n* = 19, 68.0%) (Table 4a). The majority of the women with lived/living experience never accessed sexual health services through community-based settings (*n* = 7, 30.0%). Further, the majority of women with lived/living experience accessed sexual health services less than once a year (*n* = 6, 26.0%) or 1–2 times a year (*n* = 6, 26.0%) in clinical/medical settings.

**Table 4a.** Participant Demographics for Interviews With Women With Lived/Living Experience.

Characteristics	***n***	**%**
Age		
20–30 years	3	13.0
30–40 years	3	13.0
40–50 years	9	38.0
50–60 years	5	21.0
60–70 years	4	17.0
Gender		
Cis woman	24	100.0
Lived experience as, or identifying with, the following priority groups^a^		
First Nations, Inuit, and Métis communities;	3	5.0
African, Caribbean, and Black communities and other racialized people;	5	8.0
Two-spirit people;	1	2.0
Transgender, nonbinary, or gender diverse persons	1	2.0
People who use drugs;	16	27.0
People who experience incarceration;	7	12.0
Sex workers and/or their clients;	13	22.0
People living with HIV and people with lived and living experience of hepatitis;	3	5.0
Newcomers, migrants and immigrants to Canada, particularly from regions with high HIV, hepatitis B or hepatitis C prevalence;	3	5.0
Youth	2	3.0
Prefer not to answer	1	2.0
City in which services are accessed^a^		
Toronto	19	68.0
Ottawa	5	18.0
Barrie	1	4.0
Thunder Bay	1	4.0
Saskatoon	1	4.0
Prefer not to answer	1	4.0
Frequency accessing sexual health services in a clinical/medical setting (eg physician, nurse, pharmacist, etc)?		
Never accessed sexual health services	2	9.0
Less than once a year	6	26.0
1–2 times a year	6	26.0
Once every few months	5	22.0
Once a month	2	9.0
More than once a month	2	9.0
Frequency accessing sexual health services in a community-based organization or other service provider (eg harm reduction worker, social worker, community programs coordinator, etc.)?		
Never accessed sexual health services	7	30.0
Less than once a year	1	4.0
1–2 times a year	6	26.0
Once every few months	2	9.0
Once a month	1	4.0
More than once a month	5	22.0
Other	1	4.0

a Categories were not mutually exclusive.

**Table 4b.** Participant Demographics for Interviews With Clinicians and Service Providers.

Characteristics	*n*	%
Age		
20–29 years	3	17.0
30–39 years	6	33.0
40–49 years	6	33.0
50–59 years	2	11.0
60–69 years	1	6.0
Profession/Role		
Physician	2	11.0
CBO Director	2	11.0
Registered Nurse	4	22.0
Nurse Practitioner	3	17.0
Program Research & Outreach Coordinator	3	17.0
Manager of Street Outreach, Harm Reduction, and Sexual Health Programs	1	6.0
Community Health Worker	2	11.0
Registered Midwife	1	6.0
Years working with women related to sexual health		
<5 years	5	28.0
5–10 years	3	17.0
10–15 years	3	17.0
15–20 years	4	22.0
20–25 years	1	6.0
> 25 years	2	11.0
City in which they work		
Toronto	9	50.0
Ottawa	1	6.0
Sudbury	1	6.0
Thunder Bay	5	28.0
Prince Albert	1	6.0
Saskatoon	1	6.0
Work with the following priority groups^a^		
First Nations, Inuit, and Métis communities;	12	9.0
African, Caribbean, and Black communities and other racialized people;	13	10.0
Two-spirit people;	10	8.0
Transgender, nonbinary, or gender diverse persons	14	11.0
People who use drugs;	13	10.0
People who experience incarceration;	10	8.0
Sex workers and/or their clients;	11	9.0
People living with HIV and people with lived and living experience of hepatitis;	13	10.0
Newcomers, migrants and immigrants to Canada, particularly from regions with high HIV, hepatitis B or hepatitis C prevalence;	11	9.0
Women among these populations, as appropriate	14	11.0
Youth among these populations, as appropriate	8	6.0

a Categories were not mutually exclusive.

Clinicians and service providers were typically between 30 and 49 years of age (*n* = 12, 67.0%), most often registered nurses (*n* = 4, 22.0%), and half were based in Toronto (*n* = 9, 50.0%) (Table 4b). They primarily work with people living with HIV or hepatitis (*n* = 13, 10.0%), trans, nonbinary, and gender diverse individuals (*n* = 14, 11.0%), racialized communities (*n* = 13, 10.0%), and people who use drugs (*n* = 13, 10.0%).

Thematic analysis of stakeholder interviews revealed several interconnected insights (Figure 3). Women with lived/living experience and clinicians/service providers emphasized that structural and systemic challenges—such as housing and income insecurity, limited mental health services, and racism—remain major barriers to HIV/STBBI prevention and SRH care. Many women with lived/living experience described how meeting basic needs must take precedence over preventive health, illustrating the urgency of addressing SDH within HIV prevention frameworks. Women with lived/living experience also expressed a strong desire for community connection, particularly among women at risk of HIV/STBBIs. These connections took many forms, from Indigenous women's healing through land, culture, and community to informal peer networks that shared information and supported navigation through complex systems of care.

Women with lived/living experience and clinicians/service providers highlighted the need to integrate SRH and HIV/STBBI services to minimize fragmentation across reproductive health, sexual health, and social support systems. This integration was viewed as critical to reducing missed opportunities for care and ensuring holistic, women-centred services. Several women with lived/living experience also shared that informal and accessible environments, such as drop-in centres, women's shelters, or peer groups, provided safe and trusted spaces for discussing HIV prevention. This highlights the need for HIV/STBBI prevention to extend beyond clinical settings. In addition, some women with lived/living experience began discussing PrEP, PEP, and DoxyPEP during group interviews, and many had never heard of these options before. Participants’ immediate interest in sharing this information with peers underscored the value of embedding prevention education within community-based and peer-led settings. Finally, sustainability emerged as a central concern from both women with lived/living experience and clinicians/service providers. Women with lived/living experience called for long-term, community-anchored approaches that ensure continued support and resources beyond the duration of individual studies or funding periods.

### The W-PREV Model

**Figure 4.**
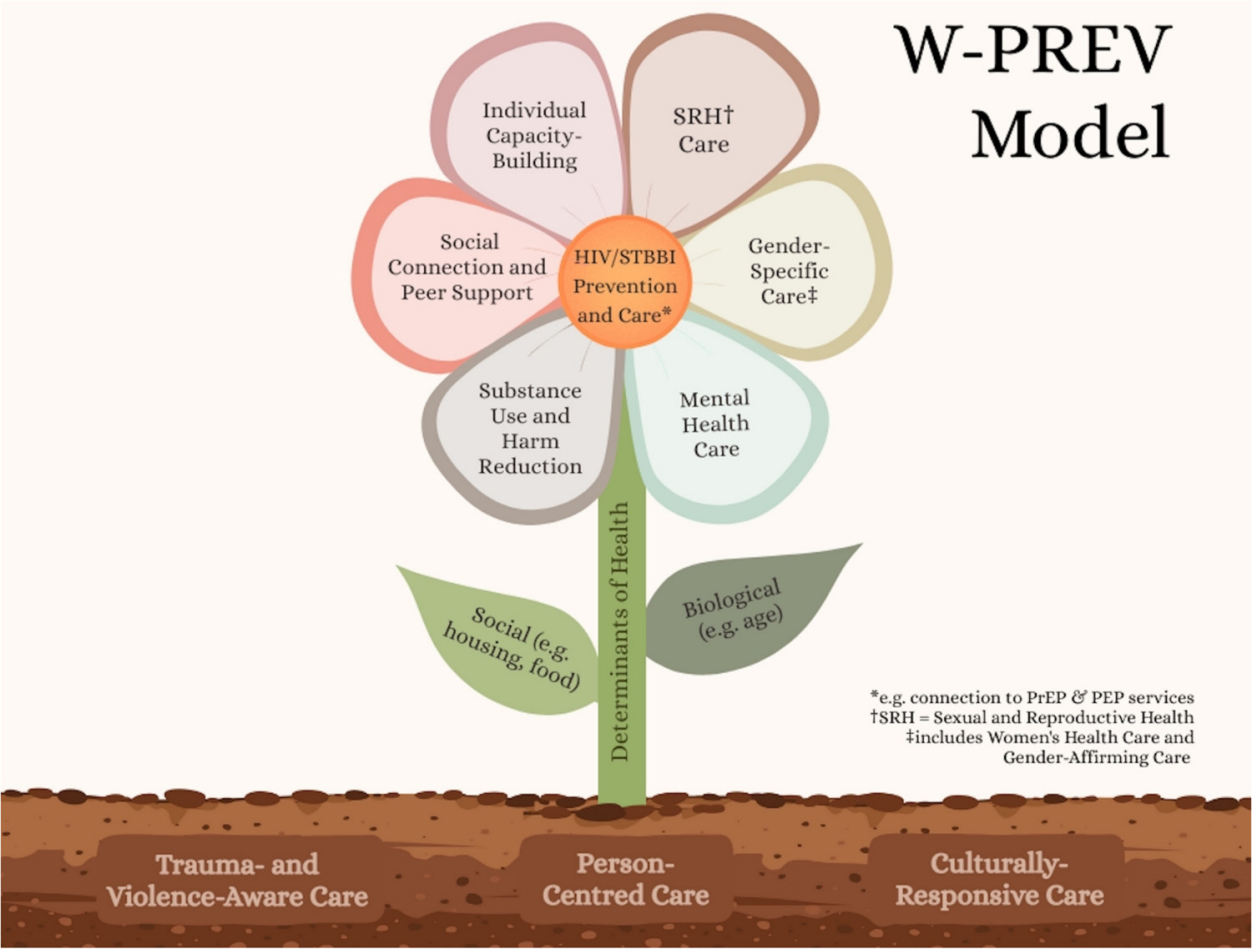
The W-PREV model. The model includes trauma- and violence-aware care, person-centred care and culturally responsive care. The stem and leaves of the flower represent social and biological determinants of health to serve as the foundation. Each petal represents a key health delivery domain that would enhance HIV/STBBI prevention and care for women: (1) sexual and reproductive health care, (2) gender-specific care, including women's health care and gender-affirming care, (3) mental health care, (4) substance use care and harm reduction, (5) social connection and peer support, and (6) individual capacity building. PrEP (pre-exposure prophylaxis); PEP (post-exposure prophylaxis).

The W-PREV Model evolved into the shape of a flower, with HIV/STBBI prevention and care at the centre and the petals symbolizing the interconnected domains of health that must work together to support the full health of women and gender diverse people (Figure 4). Each petal represents a key health delivery domain that would enhance HIV/STBBI prevention and care for women: (1) SRH care, (2) gender-specific care, including women's health care and gender-affirming care, (3) mental health care, (4) substance use care and harm reduction, (5) social connection and peer support, and (6) individual capacity building. Also essential to address, as represented by the stem and leaves, are social and biological determinants of health. The flower is nourished by the soil to represent that care must be grounded in trauma- and violence-aware, person-centred, and culturally responsive principles. The W-PREV Model uniquely applies these domains to HIV/STBBI prevention and early intervention, rather than treatment and ongoing care, and was developed directly from our environmental scan and stakeholder analyses that identified persistent prevention gaps across SRH, community supports, and system navigation.

Several frameworks underpin the W-PREV Model, with a focus on trauma- and violence-aware care (TVAC), person-centred care, and cultural responsiveness. The model is foremost grounded in TVAC, acknowledging that many women who may be at risk of acquiring HIV/STBBIs have intersecting oppressions that may have resulted in experiences of violence or trauma in their lives. In our analyses, trauma and violence repeatedly emerged as barriers to prevention engagement and early care-seeking. Therefore, integrating TVAC into W-PREV serves a preventative function by fostering trust and access before HIV/STBBIs occur. TVAC is similar to trauma-informed care (TIC) in that it aims to deliver care with an understanding that interpersonal violence and violent victimization deeply impact one's life, health and development.^44,74^ TVAC and TIC both assert that providers should attempt to dismantle power dynamics in the care environment to give the patient a sense of control and empowerment in their care and to prevent re-traumatization.^44,74^ Both frameworks strive to create safe health care environments that recognize patients’ lived experience and honor their resilience.^44,74^ TVAC is grounded in anti-oppressive, anti-colonial and harm-reductive principles, requiring providers to recognize the enduring impacts of settler colonialism and colonial violence, especially on Indigenous Peoples’ health.^44^ Still, TVAC extends on TIC by considering that “providers are not required to be experts in trauma and violence resolution, which may be implied in the term *trauma-informed*.”^44^ Employing the W-PREV Model, providers will intentionally create space for women to be heard and their choices respected, actively resisting the perpetuation of institutionalized violence.^44^

Our findings highlighted that prevention requires proactive and longitudinal engagement, shared decision-making around SRH and PrEP, and reduced care navigation barriers. These key gaps are incorporated directly into the W-PREV Model design. Person-centred care is a well-established approach with four key principles: (1) care grounded in empathy, trust, and provider competence; (2) shared decision-making and mutual responsibility between patient and care providers; (3) recognition of patient's lived experiences, expertise, and preferences; and (4) attention to the full spectrum of factors shaping health—including biological, psychological, cultural, familial, and socio-structural dimensions.^44,75–77^ Person-centred care affirms a woman's right to autonomous decision-making over her life, health, and care, but should also provide culturally-aware support, facilitating informed and confident decisions.^44,75–77^ Person-centred care also upholds that a woman's priorities/vulnerabilities will change over her life course, and having a reciprocal relationship with a care provider eases navigation through these shifts.^78^ The W-PREV Model intends to holistically examine a woman's needs, providing them with a guided and personalized action plan for longitudinal behavioral change. Further, a part of person-centred care is relational practice in health that aims to establish and maintain strong relationships and trust between patients, providers, and communities, through which positive health and well-being can be achieved.^79,80^

The focus on cultural responsiveness is prevention-driven, addressing the mistrust, stigma, racism, and exclusion that our analyses identified as major barriers to accessing HIV/STBBI prevention and SRH services. Culturally responsive care recognizes that women and gender diverse people's experiences of health, illness, and care are shaped by their intersecting social positions, histories, and relationships with colonial and institutional systems.^81–84^ The W-PREV Model integrates cultural responsiveness and the concept of intersectionality as guiding values and as practice approaches, ensuring that HIV/STBBI prevention and SRH services are not only accessible, but also affirming, relevant, and respectful of diverse positions, identities, and ways of knowing and healing. Culturally responsive care begins with knowledge and understanding of women's voices, values and viewpoints. It aims to draw on community-based customs and embed cultural safety within every interaction. This means creating space for traditional knowledge, language, and spirituality; acknowledging the intergenerational effects of racism, colonization, and gender-based violence; and creating a reciprocal dynamic with women.

Like the WCHC Model, the W-PREV Model is developed for flexibility in its delivery (eg, single or multiple providers, interdisciplinary clinics, community organizations) and settings (eg, urban, rural), with a focus on alleviating the burden of system navigation from women's responsibility. While the W-PREV Model was developed within the Canadian context, flexible delivery facilitates the tailoring of our findings within different income and resource settings. At a minimum, a community must have access to a single coordinated space where clinical (ie, HIV/STBBI testing), community, and social services can be accessed. The W-PREV Model extends the WCHC Model by applying similar principles specifically to HIV/STBBI prevention, grounded in the gaps and needs identified through our scoping review, environmental scan, and stakeholder interviews.

## Discussion

The development of the W-PREV Model was directly informed by the integrated findings of the rapid scoping review, environmental scan, and stakeholder interviews. Together, these activities highlighted major gaps in Canada's HIV/STBBI prevention landscape, specifically the dominance of biomedical approaches and the lack of attention to women's lived realities and SDH. Guided by Graham et al's Knowledge-to-Action Framework, we synthesized empirical evidence and community-based insights to adapt the WCHC Model into a prevention-focused framework responsive to women's needs. The resulting W-PREV Model highlights the need for a comprehensive, integrated, and women-centred approach that addresses the interdependence of structural, social, and clinical determinants of health. Like the WCHC Model, it integrates all components of holistic care—including SRH care, gender-specific care, including women's health care and gender-affirming care, peer support and social connection, mental health care, substance use and harm reduction, individual capacity building, and HIV/STBBI prevention and care as its central focus.

HIV/STBBI prevention strategies for women must extend beyond clinical settings and be embedded within community and social contexts. Within the W-PREV Model, this approach is operationalized through the integration of peer support and social connection as central components. These peer- and community-based spaces foster trust and safety, creating opportunities to normalize conversations around sexuality, where providers proactively and routinely discuss sexual health, dismantle power imbalances, and shift prevention from being risk-focused to empowerment-focused.^65–67^ Evidence from diverse populations, such as women who inject drugs, have shown that integrating SRH and HIV prevention in community outreach settings increases SRH service acceptability, uptake, and is predominantly preferred.^85^ Similarly, among providers and Black adolescent young girls and women, there is a strong desire to normalize sexual health conversations to reduce stigma and overcome their own biases regarding risk.^86^

The W-PREV Model reframes HIV/STBBI prevention as a holistic and empowerment-based process that situates women's experiences, relationships, and contexts at the center of care. Rather than viewing prevention as a series of biomedical interventions, the model recognizes it as part of a continuum that must integrate SRH care, harm reduction, mental health, and social support. Evidence consistently demonstrates that integrating HIV prevention within broader SRH and social-determinant–focused services leads to better outcomes, such as increased HIV and sexual health knowledge, condom use self-efficacy, and risk reduction intentions.^85,87–93^ Studies have similarly shown that integrated models of care are associated with higher HIV counseling and testing rates, increased uptake of sexual health services, and reduced stigma among women and female sex workers.^88,89,92,94^ Existing prevention efforts are often fragmented and focused in urban areas, leaving women in rural and northern regions—particularly Indigenous, racialized, and marginalized women with limited access to gender-responsive and culturally safe services. Women in rural and northern communities experience compounded disadvantages due to intersecting factors: limited health infrastructure, poverty, and social stigma.^95,96^ These factors restrict access to HIV prevention, testing, and treatment services, resulting in lower PrEP awareness and uptake, and less frequent provider-patient communication about HIV prevention options.^97–99^ The lack of nearby, integrated HIV and sexual health services forces women and gender diverse people in rural and northern areas to rely on general healthcare systems that are often overstretched and lack HIV-specific expertise or gender-responsive care, thereby limiting access to tailored prevention and support and reinforcing existing health inequities.^96,100,101^ An integrated, equitable, and adaptable approach to HIV/STBBI prevention—preferably delivered through a single, coordinated space of care where clinical, community, and social services are interconnected is needed. The W-PREV Model was designed to respond directly to these gaps by bridging clinical and community settings, integrating SRH, HIV/STBBI prevention, and social supports, and ensuring that care remains flexible, women-centred, and accessible across diverse geographic and social contexts.

The structural realities, such as racism, gender-based violence, and economic insecurity, reinforced that HIV/STBBI prevention cannot be achieved without addressing broader social and structural determinants of health. For many women, barriers such as poverty, housing and income instability, and lack of access to mental health supports often take precedence over preventive HIV health care.^102–105^ These findings affirm the W-PREV Model's foundation in the SDH and intersectionality and its integration of mental health, substance use, and harm reduction as essential domains. In this way, the W-PREV Model addresses the underlying conditions that shape prevention engagement, consistent with evidence showing that women's ability to access HIV prevention depends on addressing determinants of well-being.^106–108^

### Limitations

It is important to address that some communities may not have access to the basic care required to deliver the W-PREV Model. The model was developed within the Canadian context, specifically Ontario and Saskatchewan, and may require adaptation in regions with differing health systems, resources, or sociocultural environments. Second, the evidence base informing W-PREV draws on a rapid scoping review, environmental scan, and stakeholder interviews. Although these methods provided timely and contextually rich insights, they may not fully capture the experiences of women with limited system engagement, such as those in rural, remote, or under-resourced communities. Further, we used a rapid qualitative analysis approach for the stakeholder interviews. Without transcription, it is possible that not all experiences of interview participants were captured in our analyses, despite iterative revision of recordings and facilitator notes. Additionally, the model has not yet been quantitatively evaluated for effectiveness or reach, and implementation will require cross-sector collaboration, stable funding, and policy alignment to prevent fragmentation. In March 2026, the W-PREV Model will be operationalized into a community-based intervention in Ontario. Metrics from this pilot study will provide insights on the feasibility and sustainability of W-PREV Model implementation. Lastly, while the flower-shaped design of the W-PREV Model serves as an accessible and symbolic representation of its interconnected domains, it may not resonate with all women and will need to be further refined through user feedback and evaluation.

## Conclusions

The W-PREV Model represents a collection of knowledge from a rapid scoping review, environmental scan and interviews with women with lived/living experience and clinicians/service providers. Our goal is to genuinely respond to the needs of women and gaps in care, facilitating an urgent shift in HIV/STBBI prevention and SRH care for women in Canada. By linking HIV/STBBI prevention and SRH services with broader supports like mental health, housing, and peer supports, the W-PREV Model offers a solution that moves beyond the current biomedical approaches to HIV/STBBI prevention that produce barriers for women. The W-PREV model affirms women's agency and integrates the wisdom they have in their experiences, prioritizes flexibility and accessibility, and emphasizes individualized action planning for long-term health improvement. Further, the W-PREV Model is constructed so it may be tailored to diverse local contexts. Additional work is being done to operationalize the W-PREV Model into an adaptable, integrated, community-based intervention, consisting of operational sites, clinicians, community-outreach workers, training programs, and a needs assessment tool. The long-term aim is to integrate W-PREV within existing health and community-based programs in Canada, rather than delivering it as a standalone initiative. This includes building local capacity through peer-led care model delivery and a train-the-trainer strategy to reduce reliance on the research team over time, ensuring sustainability. A toolkit is also being developed to provide clear direction on how the W-PREV Model may be employed at various settings.

## Supplemental Material

sj-zip-1-jia-10.1177_23259582261447168 - Supplemental material for Development of the W-PREV Model: Integrating HIV/STBBI Prevention and Women's Sexual and Reproductive Healthcare Using an Intersectional Women-Centered Approach Supplemental material, sj-zip-1-jia-10.1177_23259582261447168 for Development of the W-PREV Model: Integrating HIV/STBBI Prevention and Women's Sexual and Reproductive Healthcare Using an Intersectional Women-Centered Approach by Kyla Gibson, BSc, Amy Ly, MSc, V Logan Kennedy, PhD, Angela Underhill, MSc, Stephanie Smith, Emily Bear, Cara Spence, PhD, Wangari Tharao, PhD, Molly Bannerman, MSW, Asli Mahdi, MA, Ananya Inaganti, BSc, Rsha Soud, MSc, Ashley Lacombe-Duncan, PhD, and Mona Loutfy, MD, MPH in Journal of the International Association of Providers of AIDS Care (JIAPAC)

## References

[1] Goldenberg SM Pearson J Moreheart S , et al. Prevalence and structural correlates of HIV and STI testing among a community-based cohort of women sex workers in Vancouver, Canada. Singer DE, ed. PLoS ONE. 2023;18(3):e0283729. doi:10.1371/journal.pone.0283729 PMC10062647 36996154

[2] Tazinya RMA El-Mowafi IM Hajjar JM Yaya S . Sexual and reproductive health and rights in humanitarian settings: a matter of life and death. Reprod Health. 2023;20(1):42. doi:10.1186/s12978-023-01594-z 36899344 PMC9999057

[3] Violence against women. World Health Organization. Accessed June 17, 2025. https://www.who.int/news-room/fact-sheets/detail/violence-against-women

[4] HIV in Canada: 2022 surveillance highlights. Public Health Agency of Canada. Accessed June 17, 2025. https://www.canada.ca/en/public-health/services/publications/diseases-conditions/hiv-2022-surveillance-highlights.html

[5] Challacombe L. The Epidemiology of HIV in Canada. Canada’s source for HIV and hepatitis C information. Accessed October 31, 2025. https://www.catie.ca/the-epidemiology-of-hiv-in-canada

[6] HIV and AIDS in Saskatchewan (2019). Government of Saskatchewan. Accessed October 31, 2025. https://skhiv.ca/wp-content/uploads/2020/12/HIV-in-Saskatchewan-2019-Infographic-FINAL.pdf

[7] Cozart M Magnusson D Mondal P Gartner K . Integrated prenatal care for women living with HIV: primary care outcomes in Saskatoon, Saskatchewan. J Obstet Gynaecol Can. 2022;44(5):521‐526. doi:10.1016/j.jogc.2022.01.009 35114380

[8] HIV in Canada: 2021 surveillance highlights. Public Health Agency of Canada. Accessed October 19, 2025. https://www.canada.ca/en/public-health/services/publications/diseases-conditions/hiv-2021-surveillance-highlights.html

[9] What is the prevalence of HIV in trans people? Canada’s source for HIV and hepatitis C information. Accessed October 31, 2025. https://www.catie.ca/prevention-in-focus/what-is-the-prevalence-of-hiv-in-trans-people

[10] Chlamydia, gonorrhea and infectious syphilis in Canada: 2021 surveillance data update. Public Health Agency of Canada. Accessed July 31, 2025. https://www.canada.ca/en/public-health/services/publications/diseases-conditions/chlamydia-gonorrhea-infectious-syphilis-2021-surveillance-data.html

[11] Kaur-Tiwana T Schwartz N Forbes SM , et al. Public health funding and chlamydia and gonorrhea rates among adolescents during the COVID-19 pandemic in Ontario, Canada: an interrupted time series study. Public Health. 2025;246:105845. doi:10.1016/j.puhe.2025.105845 40617107

[12] Infectious syphilis and congenital syphilis in Canada, 2021. Public Health Agency of Canada. Accessed August 4, 2025. https://www.canada.ca/en/public-health/services/reports-publications/canada-communicable-disease-report-ccdr/monthly-issue/2022-48/issue-11-12-november-december-2022/infectious-congenital-syphilis-canada-2021.html

[13] Tetteh A Abdi N Moore V Gravel G . Rising congenital syphilis rates in Canada, 1993–2022. Front Public Health. 2025;12:1522671. doi:10.3389/fpubh.2024.1522671 39897185 PMC11783095

[14] Aral SO Hawkes S Biddlecom A Padian N . Disproportionate impact of sexually transmitted diseases on women. Emerg Infect Dis. 2004;10(11):2029‐2030. doi:10.3201/eid1011.040623_02 16010734 PMC3329016

[15] Crenshaw K . Demarginalizing the intersection of race and sex: a Black feminist critique of antidiscrimination doctrine, feminist theory and antiracist politics. Routledge. 1989(1):139.

[16] Collins PH. Intersectionality as critical social theory. Duke University Press; 2019. doi:10.1215/9781478007098

[17] Vohra-Gupta S Petruzzi L Jones C Cubbin C . An intersectional approach to understanding barriers to healthcare for women. J Community Health. 2023;48(1):89‐98. doi:10.1007/s10900-022-01147-8 36273069 PMC9589537

[18] RFA-MH-19–412: Promoting Reductions in Intersectional StigMa (PRISM) to Improve the HIV Prevention Continuum (R01 Clinical Trial Optional). National Institutes of Health. November 23, 2018. https://grants.nih.gov/grants/guide/rfa-files/rfa-mh-19-412.html. Accessed January 24, 2026.

[19] Bowleg L Malekzadeh AN Mbaba M Boone CA . Ending the HIV epidemic for all, not just some: structural racism as a fundamental but overlooked social-structural determinant of the US HIV epidemic. Curr Opin HIV AIDS. 2022;17(2):40‐45. doi:10.1097/COH.0000000000000724 35102051 PMC9109814

[20] Estimates of HIV incidence, prevalence and Canada’s progress on meeting the 90-90-90 HIV targets, 2020. Public Health Agency of Canada. Accessed June 17, 2025. https://www.canada.ca/en/public-health/services/publications/diseases-conditions/estimates-hiv-incidence-prevalence-canada-meeting-90-90-90-targets-2020.html

[21] Baral SD Poteat T Strömdahl S Wirtz AL Guadamuz TE Beyrer C . Worldwide burden of HIV in transgender women: a systematic review and meta-analysis. Lancet Infect Dis. 2013;13(3):214‐222. doi:10.1016/S1473-3099(12)70315-8 23260128

[22] Lacombe-Duncan A Olawale R . Context, types, and consequences of violence across the life course: a qualitative study of the lived experiences of transgender women living with HIV. J Interpers Violence. 2022;37(5-6):2242‐2266. doi:10.1177/0886260520935093 32639854

[23] Wang SC Maher B . Substance use disorder, intravenous injection, and HIV infection: a review. Cell Transplant. 2019;28(12):1465‐1471. doi:10.1177/0963689719878380 31547679 PMC6923556

[24] Hankins C. Gender, sex, and HIV: how well are we addressing the imbalance? Curr Opin HIV AIDS. 2008;3(4):514‐520. doi:10.1097/COH.0b013e32830136b4 19373014

[25] Cohen MS . Preventing sexual transmission of HIV. Clin Infect Dis. 2007;45(Suppl. 4):S287‐S292. doi:10.1086/522552 18190301

[26] Wong T Singh A Mann J Hansen L McMahon S . Gender differences in bacterial STIs in Canada. BMC Women’s Health. 2004;4(S1):S26. doi:10.1186/1472-6874-4-S1-S26 PMC2096668 15345089

[27] Popovic N Yang Q Campeau L , et al. The prevalence of HIV pre-exposure prophylaxis (HIV-PrEP) use and HIV-PrEP-to-need ratio in nine Canadian provinces, 2018–2021. CCDR. 2025;51(1):35‐42. doi:10.14745/ccdr.v51i01a05 39781235 PMC11709143

[28] Chlamydia, gonorrhea and infectious syphilis in Canada: 2020 (infographic). Public Health Agency of Canada. Accessed June 17, 2025. https://www.canada.ca/en/public-health/services/publications/diseases-conditions/chlamydia-gonorrhea-infectious-syphilis-canada-2020-infographic.html

[29] Kalichman SC Cherry C Amaral CM , et al. Adherence to antiretroviral therapy and HIV transmission risks: implications for test-and-treat approaches to HIV prevention. AIDS Patient Care STDS. 2010;24(5):271‐277. doi:10.1089/apc.2009.0309 20438373 PMC2875951

[30] Angel JB Freilich J Arthurs E , et al. Adherence to oral antiretroviral therapy in Canada, 2010–2020. AIDS. 2023;37(13):2031‐2040. doi:10.1097/QAD.0000000000003648 37418513 PMC10552836

[31] Sewell WC Blankenship SA . Perceived HIV risk as a predictor of sexual risk behaviors and discrimination among high-risk women. AIDS Care. 2019;31(6):675‐680. doi:10.1080/09540121.2018.1533234 30318900 PMC6443477

[32] Connell E . Bridging the gap: integrating HIV prevention into sexual and reproductive health promotion. Can Woman Stud Les Cahiers De La Femme. 2001;21(2):68-71. Accessed May 31, 2025. https://cws.journals.yorku.ca/index.php/cws/article/view/12600.

[33] Trends in HIV Pre-Exposure Prophylaxis [HIV-PrEP] use in 9 Canadian provinces, 2019–2022 [Infographic]. Public Health Agency of Canada. Accessed June 18, 2025. https://www.canada.ca/en/public-health/services/publications/diseases-conditions/trends-pre-exposure-prophylaxis-use-in-9-canadian-provinces-2019-2022-infographic.html

[34] Tan DHS Hull MW Yoong D , et al. Canadian guideline on HIV pre-exposure prophylaxis and nonoccupational postexposure prophylaxis. CMAJ. 2017;189(47):E1448‐E1458. doi:10.1503/cmaj.170494 PMC5703677 29180384

[35] Namchuk AB Stranges TN Splinter TFL Moore KN Logie CH Galea LAM . Canadian Health research funding patterns for sexual and gender minority populations reflect exclusion of women. LGBT Health. 2025;12(2):144‐151. doi:10.1089/lgbt.2024.0014 38989595

[36] Loutfy M Greene S Kennedy VL , et al. Establishing the Canadian HIV women’s sexual and reproductive health cohort study (CHIWOS): operationalizing community-based research in a large national quantitative study. BMC Med Res Methodol. 2016;16(1):101, s12874-016-0190-0197. doi:10.1186/s12874-016-0190-7 27543135 PMC4992236

[37] Van Uum R Masshadi PE Canani F , et al. Characterizing cis and trans women's HIV risk and access to HIV prophylaxis in Ontario, Canada. TOAIDJ. 2025;19(1):e18746136387776. doi:10.2174/0118746136387776250825202101

[38] HIV Prevention Options for Cis and Trans Women. Women and HIV/AIDS Initiative. Accessed June 18, 2025. https://whai.ca/resource/webinar-hiv-prevention-options-for-cis-and-trans-women/

[39] PIP & Women*: What You Need To Know. Women and HIV/AIDS Initiative. Accessed June 18, 2025. https://whai.ca/wp-content/uploads/WHAI-PIP-Brochure-English-AODA.pdf

[40] Doxycycline to help prevent bacterial STIs. Canada’s source for HIV and hepatitis C information. Accessed June 18, 2025. https://www.catie.ca/sites/default/files/2024-04/fs-doxy-04162024-en.pdf

[41] Salway T Ferlatte O Shoveller J , et al. The need and desire for mental health and substance use–related services among clients of publicly funded sexually transmitted infection clinics in Vancouver, Canada. J Public Health Manag Pract. 2019;25(3):E1‐E10. doi:10.1097/PHH.0000000000000904 30444755

[42] Ross LE Sterling A Dobinson C Logie CH D’Souza S . Access to sexual and reproductive health care among young adult sex workers in Toronto, Ontario: a mixed-methods study. cmajo. 2021;9(2):E482‐E490. doi:10.9778/cmajo.20200049 PMC8157979 33990362

[43] Skerritt L De Pokomandy A O’Brien N , et al. Discussing reproductive goals with healthcare providers among women living with HIV in Canada: the role of provider gender and patient comfort. Sexual Reprod Health Matters. 2021;29(1):425‐440. doi:10.1080/26410397.2021.1932702 PMC8231384 34165395

[44] Loutfy M Tharao W Kazemi M , et al. Development of the Canadian women-centred HIV care model using the knowledge-to-action framework. J Int Assoc Provid AIDS Care. 2021;20:232595822199561. doi:10.1177/2325958221995612 PMC8047935 33845677

[45] Carter A Loutfy M De Pokomandy A , et al. Health-related quality-of-life and receipt of women-centered HIV care among women living with HIV in Canada. Women Health. 2018;58(5):498‐518. doi:10.1080/03630242.2017.1316346 28388352

[46] Koebel J Kazemi M Kennedy VL , et al. Dissemination of the women-centred HIV care model: a multimodal process and evaluation. J Int Assoc Provid AIDS Care. 2024;23:23259582231226036. doi:10.1177/23259582231226036 38389331 PMC10894538

[47] Iyer H Underhill A Persad Y , et al. Operationalizing the women-centred HIV care model for trans women and persons with transfeminine experience living with and affected by HIV: a qualitative study. Int J Transgend Health. 2025;26(4):1070‐1092. doi:10.1080/26895269.2024.2323508 41180914 PMC12573519

[48] Graham ID Logan J Harrison MB , et al. Lost in knowledge translation: time for a map? J Contin Educ Health Profess. 2006;26(1):13‐24. doi:10.1002/chp.47 16557505

[49] Arksey H O’Malley L. Scoping studies: towards a methodological framework. Int J Soc Res Methodol. 2005;8(1):19‐32. doi:10.1080/1364557032000119616

[50] Moser A Korstjens I . Series: practical guidance to qualitative research. Part 7: qualitative evidence synthesis for emerging themes in primary care research: scoping review, meta-ethnography and rapid realist review. Eur J Gen Pract. 2023;29(1):2274467. doi:10.1080/13814788.2023.2274467 37902265 PMC10990260

[51] Tricco AC Lillie E Zarin W , et al. PRISMA extension for scoping reviews (PRISMA-ScR): checklist and explanation. Ann Intern Med. 2018;169(7):467‐473. doi:10.7326/m18-0850 30178033

[52] Peters MD Godfrey C McInerney P Munn Z Tricco AC Khalil H. . Chapter 11. Scoping reviews (2020 version). In: Aromataris E Munn Z eds. JBI manual for evidence synthesis. JBI; 2020:141–146. doi:10.46658/JBIMES-20-12

[53] Morris M Boruff JT Gore GC . Scoping reviews: establishing the role of the librarian. jmla. 2017;104(4):346-353. doi:10.5195/jmla.2016.156 PMC5079503 27822163

[54] Garritty C Gartlehner G Nussbaumer-Streit B , et al. Cochrane rapid reviews methods group offers evidence-informed guidance to conduct rapid reviews. J Clin Epidemiol. 2021;130:13‐22. doi:10.1016/j.jclinepi.2020.10.007 33068715 PMC7557165

[55] Inaganti A Underhill A Lacombe-Duncan A Soud R Loutfy M . Assessing availability of HIV/sexually transmitted and blood-borne infection prevention and care services for trans women and gender diverse people in Ontario, Canada: an environmental scan of clinic websites. J Assoc Nurses AIDS Care. 2026;37(2):192-202. doi:10.1097/JNC.0000000000000609 41566536

[56] Shahid M Turin TC . Conducting comprehensive environmental scans in health research: a process for assessing the subject matter landscape: the basics of environmental scan. J Biomed Anal. 2018;1(2):71‐80. doi:10.30577/jba.2018.v1n2.13

[57] HIV diagnoses in Ontario, 2022. The Ontario HIV Epidemiology and Surveillance Initiative. Accessed October 19, 2025. https://www.ohesi.ca/wp-content/uploads/2024/11/HIV-diagnoses-in-Ontario-2022-TABLES-supplement.pdf

[58] Population: health and wellness. The Government of Saskatchewan. Accessed October 20, 2025. https://dashboard.saskatchewan.ca/health-wellness?subzone=subSouthEast4

[59] Nevedal AL Reardon CM Opra Widerquist MA , et al. Rapid versus traditional qualitative analysis using the consolidated framework for implementation research (CFIR). Implementation Sci. 2021;16(1):67. doi:10.1186/s13012-021-01111-5 PMC8252308 34215286

[60] Mullis CE Goldberg AJ Avila K Hall B Golub SA Keller MJ . Understanding attitudes of postpartum cisgender women toward integration of HIV prevention services into routine prenatal and postpartum sexual health discussions. AIDS Patient Care STDS. 2024;38(4):185‐193. doi:10.1089/apc.2023.0307 38656218 PMC11236281

[61] Church K Wringe A Lewin S , et al. Exploring the feasibility of service integration in a low-income setting: a mixed methods investigation into different models of reproductive health and HIV care in Swaziland. Deribe K, ed. PLoS One. 2015;10(5):e0126144. doi:10.1371/journal.pone.0126144 PMC4433110 25978632

[62] Decker MR Tomko C Wingo E , et al. A brief, trauma-informed intervention increases safety behavior and reduces HIV risk for drug-involved women who trade sex. BMC Public Health. 2018;18(1):e0126144. doi:10.1186/s12889-017-4624-x PMC5540183 28764681

[63] Loutfi D Andersson N Law S , et al. Can social network analysis help to include marginalised young women in structural support programmes in Botswana? A mixed methods study. Int J Equity Health. 2019;18(1):12. doi:10.1186/s12939-019-0911-8 30658637 PMC6339404

[64] Liu JX Vallin J Chiu C , et al. Designing for two: how enhancing human-centered design with behavioral nudges unlocked breakthroughs to promote young women’s psychological safety and access to reproductive care in Tanzania. Soc Sci Med. 2023;320(ut9, 8303205):115683. doi:10.1016/j.socscimed.2023.115683 36709692 PMC10798268

[65] Saleska JL Lee SJ . Normalization of preexposure prophylaxis for adolescents: empowerment, not vulnerability. JAMA Pediatr. 2020;174(12):1133‐1134. doi:10.1001/jamapediatrics.2020.2545 32804198 PMC8302259

[66] Zhang C Fiscella K Liu Y . Exploring the role of provider–patient communication in women’s sexual health and Pre-exposure prophylaxis care in the primary care settings in New York state of the United States. Int J Environ Res Public Health. 2022;19(13):8084. doi:10.3390/ijerph19138084 35805743 PMC9265266

[67] Hill MJ Sophus AI Sapp S Campbell J Santa Maria D Stockman JK . Examining perceptions among healthcare providers on their awareness of and experience with prescribing and/or referring pre-exposure prophylaxis to eligible cisgender black female patients: a qualitative inquiry. Int J Environ Res Public Health. 2025;22(3):450. doi:10.3390/ijerph22030450 40238560 PMC11941890

[68] Patrão AL McIntyre TM Costa ECV Matediane E Azevedo V . Testing the effectiveness of two psychosocial interventions on socio-cognitive risk factors for HIV/AIDS in Mozambican women: a randomized controlled trial. AIDS Educ Prev. 2021;33(3):169‐186. doi:10.1521/aeap.2021.33.3.169 34014113

[69] Alexander KA Jemmott LS Teitelman AM D’Antonio P. Addressing sexual health behaviour during emerging adulthood: a critical review of the literature. J Clin Nurs. 2015;24(1-2):4‐18. doi:10.1111/jocn.12640 24988875 PMC5565392

[70] Drummond PD Mizan A Brocx K Wright B . Using peer education to increase sexual health knowledge among West African refugees in Western Australia. Health Care Women Int. 2011;32(3):190‐205. doi:10.1080/07399332.2010.529215 21337242

[71] Bose DL Hundal A Singh S , et al. Evidence and gap map report: social and behavior change communication (SBCC) interventions for strengthening HIV prevention and research among adolescent girls and young women (AGYW) in low- and middle-income countries (LMICs). Campbell Syst Rev. 2023;19(1):e1297. doi:10.1002/cl2.1297 PMC9831290 36911864

[72] Logie CH Lys CL Mackay K MacNeill N Pauchulo A Yasseen AS . Syndemic factors associated with safer sex efficacy among northern and Indigenous adolescents in Arctic Canada. IntJ Behav Med. 2019;26(4):449‐453. doi:10.1007/s12529-019-09797-0 31218560

[73] Kanekar AS . HIV/AIDS counseling skills and strategies: can testing and counseling curb the epidemic? Int J Prev Med. 2011;2(1):10‐14. 21448398 PMC3063469

[74] Elliott DE Bjelajac P Fallot RD Markoff LS Reed BG . Trauma-informed or trauma-denied: principles and implementation of trauma-informed services for women. J Commun Psychol. 2005;33(4):461‐477. doi:10.1002/jcop.20063

[75] Scholl I Zill JM Härter M Dirmaier J. An integrative model of patient-centeredness – a systematic review and concept analysis. Wu WCH, ed. PLoS One. 2014;9(9):e107828. doi:10.1371/journal.pone.0107828 PMC4168256 25229640

[76] Hudon C Fortin M Haggerty JL Lambert M Poitras ME . Measuring patients’ perceptions of patient-centered care: a systematic review of tools for family medicine. Ann Family Med. 2011;9(2):155‐164. doi:10.1370/afm.1226 PMC3056864 21403143

[77] Leplege A Gzil F Cammelli M Lefeve C Pachoud B Ville I . Person-centredness: conceptual and historical perspectives. Disabil Rehabil. 2007;29(20–21):1555‐1565. doi:10.1080/09638280701618661 17922326

[78] Starfield B . Is patient-centered care the same as person-focused care? TPJ. 2011;15(2):63‐69. doi:10.7812/TPP/10-148 PMC3140752 21841928

[79] Lamph G Nowland R Boland P , et al. Relational practice in health, education, criminal justice, and social care: a scoping review. Syst Rev. 2023;12(1):194. doi:10.1186/s13643-023-02344-9 37833785 PMC10571424

[80] Pinto RM Chen Y Park S(E . A client-centered relational framework on barriers to the integration of HIV and substance use services: a systematic review. Harm Reduct J. 2019;16(1):71. doi:10.1186/s12954-019-0347-x 31856845 PMC6923912

[81] Barr E Marshall LJ Collins LF , et al. Centring the health of women across the HIV research continuum. Lancet HIV. 2024;11(3):e186‐e194. doi:10.1016/S2352-3018(24)00004-3 PMC11301651 38417977

[82] Hassan KS Coon DW Uriri-Glover J McCarthy M . Positive influences: how provider actions affect HIV care engagement for black women in the southwest U.S. Int J Environ Res Public Health. 2025;22(9):1319. doi:10.3390/ijerph22091319 41007462 PMC12469552

[83] O’Connor BB . Promoting cultural competence in HIV/AIDS care. J Assoc Nurs AIDS Care. 1996;7:41‐53. doi:10.1016/S1055-3290(96)80006-9 9021643

[84] Vu M Enders M Evans DP , et al. ‘You want to treat all patients the same. . .but it’s important to know where someone is coming from’: a qualitative study of U.S. healthcare providers’ perspectives on culturally relevant sexual and reproductive healthcare for refugee women. Health Educ Res. 2025;40(4):cyaf032. doi:10.1093/her/cyaf032 PMC12342876 40795935

[85] Ayon S Jeneby F Hamid F Badhrus A Abdulrahman T Mburu G . Developing integrated community-based HIV prevention, harm reduction, and sexual and reproductive health services for women who inject drugs. Reprod Health. 2019;16(S1):59. doi:10.1186/s12978-019-0711-z 31138238 PMC6538559

[86] Hill SV Pratt MC Elopre L Simpson T Gaines Lanzi R Matthews LT . “Nobody wants to have conversation about HIV.” A thematic analysis of in-depth interviews with black adolescent women and providers about strategies for discussing sexual health and HIV prevention. Sex Transm Dis. 2024;51(7):466‐471. doi:10.1097/OLQ.0000000000001972 38597652 PMC11182704

[87] Bulstra CA Hontelez JAC Otto M , et al. Integrating HIV services and other health services: a systematic review and meta-analysis. Nosyk B, ed. PLoS Med. 2021;18(11):e1003836. doi:10.1371/journal.pmed.1003836 PMC8577772 34752477

[88] Hully A Mallah R Villa G Gilleece Y . Integrating services to improve quality of care for women living with HIV: a global systematic review. HIV Med. 2022;23(4):310‐318. doi:10.1111/hiv.13258 35212105

[89] Shahmanesh M Chimbindi N Busang J , et al. Effectiveness of integrating HIV prevention within sexual reproductive health services with or without peer support among adolescents and young adults in rural KwaZulu-Natal, South Africa (Isisekelo Sempilo): 2 × 2 factorial, open-label, randomised controlled trial. Lancet HIV. 2024;11(7):e449‐e460. doi:10.1016/S2352-3018(24)00119-X 38925731

[90] Sikkema KJ Neufeld SA Hansen NB , et al. Integrating HIV prevention into services for abused women in South Africa. AIDS Behav. 2010;14(2):431‐439. doi:10.1007/s10461-009-9620-4 19826941 PMC3249384

[91] Ports KA Haffejee F Mosavel M Rameshbabu A . Integrating cervical cancer prevention initiatives with HIV care in resource-constrained settings: a formative study in Durban, South Africa. Glob Public Health. 2015;10(10):1238‐1251. doi:10.1080/17441692.2015.1008021 25654190 PMC4526453

[92] Narasimhan M Yeh PT Haberlen S Warren CE Kennedy CE . Integration of HIV testing services into family planning services: a systematic review. Reprod Health. 2019;16(S1):61. doi:10.1186/s12978-019-0714-9 31138307 PMC6538541

[93] Kiruthu-Kamamia C Viola E Sande O , et al. Integrating gender-based violence services into HIV care: insights from Malawi. Glob Health Sci Pract. 2025;13(1):e2400177. doi:10.9745/GHSP-D-24-00177 PMC12352945 40813243

[94] Kangudie DM Guidigbi H Mensah S Bala AA Delate R. Effective integration of sexual reproductive health and HIV prevention, treatment, and care services across Sub-Saharan Africa: where is the evidence for program implementation? Reprod Health. 2019;16(S1):56. doi:10.1186/s12978-019-0709-6 31138223 PMC6538537

[95] Jaworsky D Logie CH Wagner AC , et al. Geographic differences in the experiences of HIV-related stigma for women living with HIV in northern and rural communities of Ontario, Canada. Rural Remote Health. 2018;18(3):1‐14. doi:10.22605/RRH4522 30037269

[96] Golestani R Farahani FK Peters P . Exploring barriers to accessing health care services by young women in rural settings: a qualitative study in Australia, Canada, and Sweden. BMC Public Health. 2025;25(1):213. doi:10.1186/s12889-025-21387-2 39825291 PMC11742782

[97] Williams PA Uhrig JD Zulkiewicz BA Johnson M Anderson SKE August EM . Differences between rural and urban America that inform HIV prevention messaging. AIDS Behav. 2025;29(8):2496‐2508. doi:10.1007/s10461-025-04710-1 40327269

[98] Endalamaw A Gilks CF Ambaw F Shiferaw WS Assefa Y . Explaining inequity in knowledge, attitude, and services related to HIV/AIDS: a systematic review. BMC Public Health. 2024;24(1):1815. doi:10.1186/s12889-024-19329-5 38978024 PMC11229290

[99] Schafer KR Albrecht H Dillingham R , et al. The continuum of HIV care in rural communities in the United States and Canada: what is known and future research directions. JAIDS J Acquir Immune Defic Syndr. 2017;75(1):35‐44. doi:10.1097/QAI.0000000000001329 28225437 PMC6169533

[100] Merrell MA Crouch E Harrison S Brown MJ Brown T Pearson WS . Identifying the need for and availability of evidence-based care for sexually transmitted infections in rural primary care clinics. Sexual Trans Dis. 2024;51(2):96‐101. doi:10.1097/OLQ.0000000000001901 PMC11726064 37963336

[101] Bono RS Dahman B Sabik LM , et al. Human immunodeficiency virus–experienced clinician workforce capacity: urban–rural disparities in the southern United States. Clin Infect Dis. 2021;72(9):1615‐1622. doi:10.1093/cid/ciaa300 32211757 PMC8096280

[102] Leddy AM Zakaras JM Shieh J , et al. Intersections of food insecurity, violence, poor mental health and substance use among US women living with and at risk for HIV: evidence of a syndemic in need of attention. Lima VD, ed. PLoS One. 2021;16(5):e0252338. doi:10.1371/journal.pone.0252338 PMC8153505 34038490

[103] KabirP ShannonK KestlerM ThompsonC ZhouH DeeringK . Socio-structural barriers to mental health services among women living with HIV in metro Vancouver, Canada. AIDS Care. 2025;37(8):1221‐1232. doi:10.1080/09540121.2025.253411540823718

[104] Park E Stockman JK Thrift B Smith NA R L . Structural barriers to women’s sustained engagement in HIV care in southern California. AIDS Behav. 2020;24(10):2966‐2974. doi:10.1007/s10461-020-02847-9 32323105 PMC7790164

[105] Lee K Trujillo L Olansky E , et al. Factors associated with use of HIV prevention and health care among transgender women — seven urban areas, 2019–2020. MMWR Morb Mortal Wkly Rep. 2022;71(20):673‐679. doi:10.15585/mmwr.mm7120a1 35588092 PMC9129907

[106] Rimmler S Golin C Coleman J , et al. Structural barriers to HIV prevention and services: perspectives of African American women in low-income communities. Health Educ Behav. 2022;49(6):1022‐1032. doi:10.1177/10901981221109138 35856333 PMC9574897

[107] Logie CH Jenkinson JIR Earnshaw V Tharao W Loutfy MR. A structural equation model of HIV-related stigma, racial discrimination, housing insecurity and wellbeing among African and Caribbean black women living with HIV in Ontario, Canada. Faragher EB, ed. PLoS One. 2016;11(9):e0162826. doi:10.1371/journal.pone.0162826 PMC5036880 27669510

[108] Sharma R Dale SK . Using network analysis to assess the effects of trauma, psychosocial, and socioeconomic factors on health outcomes among black women living with HIV. AIDS Behav. 2023;27(2):400‐415. doi:10.1007/s10461-022-03774-7 35927538 PMC10712664

