# Supplementary material for: Development of the W-PREV Model: Integrating HIV/STBBI Prevention and Women's Sexual and Reproductive Healthcare Using an Intersectional Women-Centered Approach
Source: J Int Assoc Provid AIDS Care. 2026 May 8;25:23259582261447168. doi: 10.1177/23259582261447168 (PMC13167292; doi:10.1177/23259582261447168)
Supplement: sj-zip-1-jia-10.1177_23259582261447168 - Supplemental material for Development of the W-PREV Model: Integrating HIV/STBBI Prevention and Women's Sexual and Reproductive Healthcare Using an Intersectional Women-Centered Approach [file sj-zip-1-jia-10.1177_23259582261447168.zip › Supplementary Table 5.docx]

| Service Type | Far North | North Central West | North Central East | Saskatoon | South West | South East | Regina | Total Saskatchewan |
| --- | --- | --- | --- | --- | --- | --- | --- | --- |
|  | **n (%)** | **n (%)** | **n (%)** | **n (%)** | **n (%)** | **n (%)** | **n (%)** | **n (%)** |
| Women's services | 0 (0.0) | 1 (50.0) | 0 (0.0) | 5 (100.0) | 1 (100.0) | 1 (100.0) | 4 (66.7) | 12 (80.0) |
| Services addressing SDH | 0 (0.0) | 1 (50.0) | 0 (0.0) | 4 (80.0) | 0 (0.0) | 0 (0.0) | 1 (16.7) | 6 (40.0) |

**Supplementary Table 5.** Other services at clinics offering STBBI prevention services in Saskatchewan. STBBI (sexually transmitted and blood-borne infections); SDH (social determinants of health).
