# Supplementary material for: Development of the W-PREV Model: Integrating HIV/STBBI Prevention and Women's Sexual and Reproductive Healthcare Using an Intersectional Women-Centered Approach
Source: J Int Assoc Provid AIDS Care. 2026 May 8;25:23259582261447168. doi: 10.1177/23259582261447168 (PMC13167292; doi:10.1177/23259582261447168)
Supplement: sj-zip-1-jia-10.1177_23259582261447168 - Supplemental material for Development of the W-PREV Model: Integrating HIV/STBBI Prevention and Women's Sexual and Reproductive Healthcare Using an Intersectional Women-Centered Approach [file sj-zip-1-jia-10.1177_23259582261447168.zip › Supplementary Table 6.docx]

| **Data Type** | **Toronto** | **Ottawa** | **Northern** | **Eastern** | **Central East** | **Central West** | **South West** |
| --- | --- | --- | --- | --- | --- | --- | --- |
| Population^a^ | 3025647 | 1071868 | 819744 | 907777 | 4550794 | 2960636 | 1772950 |
| Number of clinics | 40 | 13 | 16 | 9 | 32 | 37 | 19 |
| Per capita | 0.00001322 | 0.00001213 | 0.00001952 | 0.00000991 | 0.00000703 | 0.00001250 | 0.00001072 |
| Per 100,000 | 1.32 | 1.21 | 1.95 | 0.99 | 0.70 | 1.25 | 1.07 |

**Supplementary Table 6.** Clinics offering STBBI prevention services per 100,000 people in Ontario by health region. ^a^Data adapted from a publicly available data source.^57^
