# Supplementary material for: Development of the W-PREV Model: Integrating HIV/STBBI Prevention and Women's Sexual and Reproductive Healthcare Using an Intersectional Women-Centered Approach
Source: J Int Assoc Provid AIDS Care. 2026 May 8;25:23259582261447168. doi: 10.1177/23259582261447168 (PMC13167292; doi:10.1177/23259582261447168)
Supplement: sj-zip-1-jia-10.1177_23259582261447168 - Supplemental material for Development of the W-PREV Model: Integrating HIV/STBBI Prevention and Women's Sexual and Reproductive Healthcare Using an Intersectional Women-Centered Approach [file sj-zip-1-jia-10.1177_23259582261447168.zip › Supplementary Table 7.docx]

| **Data Type** | **Far North** | **North Central West** | **North Central East** | **Saskatoon** | **South West** | **South East** | **Regina** |
| --- | --- | --- | --- | --- | --- | --- | --- |
| Population^a^ | 38959 | 100138 | 130551 | 338106 | 136091 | 187662 | 273351 |
| Number of clinics | 0 | 2 | 0 | 5 | 1 | 1 | 6 |
| Per capita | 0.00000000 | 0.00001997 | 0.00000000 | 0.00001479 | 0.00000735 | 0.00000533 | 0.00002195 |
| Per 100,000 | 0.00 | 2.00 | 0.00 | 1.48 | 0.73 | 0.53 | 2.19 |

**Supplementary Table 7.** Clinics offering STBBI prevention services per 100,000 people in Saskatchewan by health region. ^a^Data adapted from a publicly available data source.^58^
