# Supplementary material for: Development of the W-PREV Model: Integrating HIV/STBBI Prevention and Women's Sexual and Reproductive Healthcare Using an Intersectional Women-Centered Approach
Source: J Int Assoc Provid AIDS Care. 2026 May 8;25:23259582261447168. doi: 10.1177/23259582261447168 (PMC13167292; doi:10.1177/23259582261447168)
Supplement: sj-zip-1-jia-10.1177_23259582261447168 - Supplemental material for Development of the W-PREV Model: Integrating HIV/STBBI Prevention and Women's Sexual and Reproductive Healthcare Using an Intersectional Women-Centered Approach [file sj-zip-1-jia-10.1177_23259582261447168.zip › Appendix 1.docx]

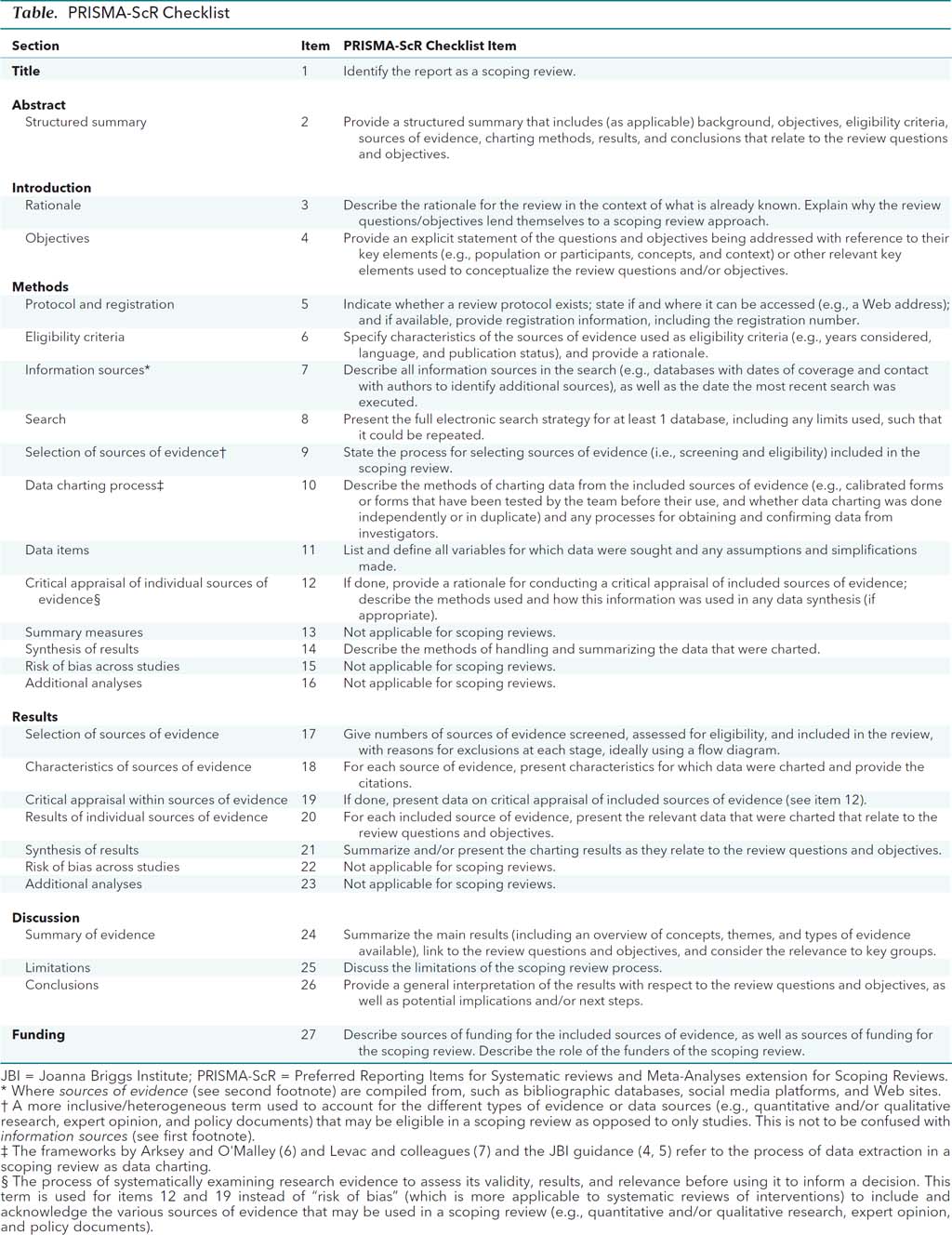


**Appendix 1.** PRISMA Extension for Scoping Reviews Checklist. Data adapted from Tricco AC, Lillie E, Zarin W, et al. PRISMA Extension for Scoping Reviews (PRISMA-ScR): Checklist and Explanation. Ann Intern Med. 2018;169(7):467-473. doi:10.7326/m18-0850
