# Supplementary material for: Development of the W-PREV Model: Integrating HIV/STBBI Prevention and Women's Sexual and Reproductive Healthcare Using an Intersectional Women-Centered Approach
Source: J Int Assoc Provid AIDS Care. 2026 May 8;25:23259582261447168. doi: 10.1177/23259582261447168 (PMC13167292; doi:10.1177/23259582261447168)
Supplement: sj-zip-1-jia-10.1177_23259582261447168 - Supplemental material for Development of the W-PREV Model: Integrating HIV/STBBI Prevention and Women's Sexual and Reproductive Healthcare Using an Intersectional Women-Centered Approach [file sj-zip-1-jia-10.1177_23259582261447168.zip › Appendix 2.docx]

**CONSULTATION GUIDE A: Women with lived/living experience**

**Social and Structural Determinants of Health**

1. What do you think women struggle with in your community?
2. Where can women turn if they’re in need of assistance?
3. What forms of women’s health support are available in your community?
   1. What services or organizations are available specifically to women in your community?
   2. What services or organizations do women access the most in their communities?

**SRH-Specific Health Needs, Experiences, and Priorities**

1. What are the sexual and reproductive health (SRH) needs that you think women have?
2. What are the women specific sexual health services in your community?
   1. Where do you think women go for SRH services?
   2. Where do women receive their SRH information/knowledge?
3. What is missing in your community for women to have their SRH needs met? Consider what improvements are needed holistically, what changes would increase access to sexual health services, and who might support or oppose these changes.
4. What’s the dream SRH care experience? Think about who is providing the services, what services are available, and how it connects to other aspects of one’s life and health.

**CONSULTATION GUIDE B: Clinicians/service providers**

**Social and Structural Determinants of Health**

1. What do you think women struggle with in your community?
2. Where can women turn if they’re in need of assistance?
3. What forms of women’s health support are available in your community?
   1. What services or organizations are available specifically to women in your community?
   2. What services or organizations do women access the most in their communities?

**Care Provider Experiences (if relevant)**

1. Do you provide SRH services in your role?
   1. If yes, can you tell us more about your relationship with women accessing this care?
      1. Probes include: did you know them before, why are they accessing care, what services/care do you offer, do you see them on an ongoing basis?

**SRH-Specific Health Needs**

1. What are the SRH needs that you think women have?
2. What groups of women in your community face barriers to accessing sexual health services in your community?
   1. What are the barriers?
3. What are the women-specific sexual health services in your community?
   1. Where do you think women go for SRH services?
   2. Where do women receive their SRH information/knowledge?
4. What is missing in your community for women to have their SRH needs met?
   1. Consider what improvements are needed holistically, what changes would increase access to sexual health services, and who might support or oppose these changes.
5. As a provider, what is the ideal environment in which to provide SRH care? Think about who is part of that team, what services are available/integrated, what are the ideal ways CBOs/community workers can be engaged in this kind of work, and how do they connect to other aspects of women’s life and health?
