# Supplementary material for: Development of the W-PREV Model: Integrating HIV/STBBI Prevention and Women's Sexual and Reproductive Healthcare Using an Intersectional Women-Centered Approach
Source: J Int Assoc Provid AIDS Care. 2026 May 8;25:23259582261447168. doi: 10.1177/23259582261447168 (PMC13167292; doi:10.1177/23259582261447168)
Supplement: sj-zip-1-jia-10.1177_23259582261447168 - Supplemental material for Development of the W-PREV Model: Integrating HIV/STBBI Prevention and Women's Sexual and Reproductive Healthcare Using an Intersectional Women-Centered Approach [file sj-zip-1-jia-10.1177_23259582261447168.zip › Supplementary Table 2.docx]

| Prevention Type | Toronto | Ottawa | Northern | Eastern | Central East | Central West | South West | Total Ontario |
| --- | --- | --- | --- | --- | --- | --- | --- | --- |
|  | **n (%)** | **n (%)** | **n (%)** | **n (%)** | **n (%)** | **n (%)** | **n (%)** | **n (%)** |
| STBBI testing | 25 (62.5) | 9 (69.2) | 14 (87.5) | 9 (100.0) | 29 (90.6) | 31 (83.8) | 16 (84.2) | 133 (80.1) |
| DoxyPEP | 3 (7.5) | 0 (0.0) | 0 (0.0) | 0 (0.0) | 0 (0.0) | 0 (0.0) | 0 (0.0) | 3 (1.8) |
| Safer sex supplies | 20 (50.0) | 4 (30.8) | 8 (50.0) | 4 (44.4) | 21 (65.6) | 18 (48.6) | 12 (63.2) | 87 (52.4) |
| Mpox vaccination | 1 (2.5) | 1 (7.7) | 2 (12.5) | 0 (0.0) | 5 (15.6) | 4 (10.8) | 1 (5.3) | 14 (8.4) |
| HPV vaccination | 9 (22.5) | 4 (30.8) | 5 (31.3) | 1 (11.1) | 8 (25.0) | 15 (40.5) | 3 (15.8) | 45 (27.1) |
| Hep A or B vaccination | 10 (25.0) | 4 (30.8) | 8 (50.0) | 2 (22.2) | 8 (25.0) | 13 (35.1) | 4 (21.1) | 49 (29.5) |
| Pap testing (cervical) | 8 (20.0) | 3 (23.1) | 7 (43.8) | 2 (22.2) | 14 (43.8) | 10 (27.0) | 9 (47.4) | 53 (31.9) |
| Pap testing (anal) | 1 (2.5) | 0 (0.0) | 0 (0.0) | 1 (11.1) | 0 (0.0) | 0 (0.0) | 0 (0.0) | 2 (1.2) |
| Sexual health counselling | 7 (17.5) | 1 (7.7) | 2 (12.5) | 1 (11.1) | 6 (18.8) | 3 (8.1) | 5 (26.3) | 25 (15.1) |
| Harm reduction | 18 (45.0) | 4 (30.8) | 12 (75.0) | 5 (55.6) | 8 (25.0) | 10 (27.0) | 16 (84.2) | 73 (44.0) |

**Supplementary Table 2.** Number of clinics offering each STBBI prevention service in Ontario.

STBBI (sexually transmitted and blood-borne infections); DoxyPEP (doxycyline post-exposure prophylaxis).
