# Supplementary material for: Development of the W-PREV Model: Integrating HIV/STBBI Prevention and Women's Sexual and Reproductive Healthcare Using an Intersectional Women-Centered Approach
Source: J Int Assoc Provid AIDS Care. 2026 May 8;25:23259582261447168. doi: 10.1177/23259582261447168 (PMC13167292; doi:10.1177/23259582261447168)
Supplement: sj-zip-1-jia-10.1177_23259582261447168 - Supplemental material for Development of the W-PREV Model: Integrating HIV/STBBI Prevention and Women's Sexual and Reproductive Healthcare Using an Intersectional Women-Centered Approach [file sj-zip-1-jia-10.1177_23259582261447168.zip › Supplementary Table 3.docx]

| **Prevention Type** | **Far North** | **North Central West** | **North Central East** | **Saskatoon** | **South West** | **South East** | **Regina** | **Total Saskatchewan** |
| --- | --- | --- | --- | --- | --- | --- | --- | --- |
|  | **n (%)** | **n (%)** | **n (%)** | **n (%)** | **n (%)** | **n (%)** | **n (%)** | **n (%)** |
| STBBI testing | 0 (0.0) | 2 (100.0) | 0 (0.0) | 4 (80.0) | 1 (100.0) | 1 (100.0) | 4 (66.7) | 12 (80.0) |
| DoxyPEP | 0 (0.0) | 0 (0.0) | 0 (0.0) | 0 (0.0) | 0 (0.0) | 0 (0.0) | 0 (0.0) | 0 (0.0) |
| Safer sex supplies | 0 (0.0) | 2 (100.0) | 0 (0.0) | 1 (20.0) | 0 (0.0) | 0 (0.0) | 2 (33.3) | 5 (33.3) |
| HPV vaccination | 0 (0.0) | 1 (50.0) | 0 (0.0) | 1 (20.0) | 0 (0.0) | 0 (0.0) | 2 (33.3) | 4 (26.7) |
| Hep A or B vaccination | 0 (0.0) | 0 (0.0) | 0 (0.0) | 0 (0.0) | 0 (0.0) | 0 (0.0) | 0 (0.0) | 0 (0.0) |
| Pap testing (cervical) | 0 (0.0) | 0 (0.0) | 0 (0.0) | 3 (60.0) | 1 (100.0) | 1 (100.0) | 3 (50.0) | 8 (53.3) |
| Pap testing (anal) | 0 (0.0) | 0 (0.0) | 0 (0.0) | 0 (0.0) | 0 (0.0) | 0 (0.0) | 0 (0.0) | 0 (0.0) |
| Sexual health counselling | 0 (0.0) | 0 (0.0) | 0 (0.0) | 0 (0.0) | 0 (0.0) | 0 (0.0) | 1 (16.7) | 1 (6.7) |
| Harm reduction | 0 (0.0) | 1 (50.0) | 0 (0.0) | 2 (40.0) | 0 (0.0) | 0 (0.0) | 2 (33.3) | 5 (33.3) |

**Supplementary Table 3.** Number of clinics offering each STBBI prevention service in Saskatchewan. STBBI (sexually transmitted and blood-borne infections); DoxyPEP (doxycyline post-exposure prophylaxis).
