# Supplementary material for: Development of the W-PREV Model: Integrating HIV/STBBI Prevention and Women's Sexual and Reproductive Healthcare Using an Intersectional Women-Centered Approach
Source: J Int Assoc Provid AIDS Care. 2026 May 8;25:23259582261447168. doi: 10.1177/23259582261447168 (PMC13167292; doi:10.1177/23259582261447168)
Supplement: sj-zip-1-jia-10.1177_23259582261447168 - Supplemental material for Development of the W-PREV Model: Integrating HIV/STBBI Prevention and Women's Sexual and Reproductive Healthcare Using an Intersectional Women-Centered Approach [file sj-zip-1-jia-10.1177_23259582261447168.zip › Supplementary Table 4.docx]

| **Service Type** | **Toronto** | **Ottawa** | **Northern** | **Eastern** | **Central East** | **Central West** | **South West** | **Total Ontario** |
| --- | --- | --- | --- | --- | --- | --- | --- | --- |
|  | **n (%)** | **n (%)** | **n (%)** | **n (%)** | **n (%)** | **n (%)** | **n (%)** | **n (%)** |
| Women's services | 29 (72.5) | 4 (30.8) | 9 (56.3) | 7 (77.8) | 24 (75.0) | 31 (83.8) | 16 (84.2) | 120 (72.3) |
| Services addressing SDH | 28 (70.0) | 4 (30.8) | 7 (43.8) | 3 (33.3) | 6 (18.8) | 13 (35.1) | 5 (26.3) | 66 (39.8) |
| Services for sex workers | 9 (22.5) | 2 (15.4) | 1 (6.3) | 0 (0.0) | 0 (0.0) | 8 (21.6) | 0 (0.0) | 20 (12.0) |

**Supplementary Table 4.** Other services at clinics offering STBBI prevention services in Ontario. STBBI (sexually transmitted and blood-borne infections); SDH (social determinants of health).
